# An AI-driven model for risk stratification and prototype intervention mapping in motoric cognitive risk syndrome and subjective cognitive decline

**DOI:** 10.3389/fnagi.2026.1878389

**Published:** 2026-10-23

**Authors:** Zhihan Wang, Dingying Liu, Bosi Zhou, Shuo Chen, Qing Xu, Haonan Ding, Bing Xu, Xue Qin

**Affiliations:** Department of Neurology, Chest Hospital of Shenyang Pharmaceutical University, Shenyang, Liaoning, China

**Keywords:** improved light spectrum optimizer, motoric cognitive risk syndrome, multimodal CNN fusion, personalized intervention mapping, risk stratification

## Abstract

As Alzheimer's disease and other dementias continue their global rise, there is a compelling need for early identification of pre-clinical cognitive decline. Motoric Cognitive Risk Syndrome and Subjective Cognitive Decline represent crucial transition phases to facilitate targeted intervention, and existing unimodal approaches are neither sensitive nor sufficiently multimodal for appropriate risk classification and individualized intervention mapping (prototype). This study introduces a hybrid multimodal CNN fusion framework based on an improved version of light spectrum optimizer as metaheuristic optimizer to address this challenge. The framework fuses multiple modality-specific information through parallel three-dimensional convolutional neural network branches (MRI and PET) and encoders of motor, cognitive, and biomarker information followed by a combination of intermediate and late fusion layers with parameters tuned by the optimized framework. The framework is meticulously assessed on the BIOCARD longitudinally dataset and achieved an accuracy of 93.7% and an area under the ROC curve (AUC) of 0.965. It significantly surpassed seven state-of-the-art methods and revealed clinical interpretability through longitudinal risk trajectories and SHAP attribution of features.

## 1 Introduction

With the rapidly increasing burden of Alzheimer's disease and related dementias (ADRD) worldwide, there is a pressing need for early detection of high-risk individuals in the early pre-clinical phase of cognitive decline (Masurkar et al., 2024). Motoric Cognitive Risk Syndrome (MCR) (Meiner et al., 2020) and Subjective Cognitive Decline (SCD) (Zhou et al., 2025) are two transitional syndromes that indicate elevated risk years prior to objective cognitive decline or functional loss. MCR is defined clinically by concurrent slow gait (usually defined as gait speed one standard deviation below age- and sex-adjusted means) and subjective cognitive complaint, in the absence of dementia, serious mobility disability, or objective cognitive deficit by standardized neuropsychological testing (Adkins-Jackson et al., 2023). SCD refers to the individual's perception of cognitive decline, particularly for memory functions, which appears disproportionate for normal aging and is not accounted for by observed cognitive deficits.

Both entities are now well-accepted to be significant predictors in the earliest part of the continuum of cognitive aging. In population-based studies, the combined prevalence estimates for MCR among community-dwelling adults aged 60 and older have been estimated to be approximately 9%−10%, from 7.3% in large cohorts of Chinese aged individuals to 10.7% in a Latin American population, have been shown to increase with age, and are higher in women (Islam et al., 2024).

The prevalence of SCD is even higher in this population and will occur between 11 and 26% of the elderly depending on measurement instruments and population group studied (prevalence was 11.1%, or one in nine in U.S. subjects aged 45 and older as determined by CDC and will be higher in enriched cohorts) (Kuhn et al., 2025). In longitudinal studies, the MCR individual is associated with an approximately 2-fold higher risk for the development of incident cognitive impairment and dementia whereas individuals with SCD have similarly higher risk (HR usually ranges from 1.5 to 3.0 for progression to MCI or ADRD).

Thus, these syndromes hold the promise of being windows of opportunity for early intervention because of the presence of commonly interacting and contributing vascular, sedentary, depression, and inflammation factors, which may promote underlying neurodegenerative and/or cerebrovascular diseases.

In spite of their prognostic value, current diagnostic methods for MCR and SCD have a number of limitations. The use of single-modality assessments (e.g., standalone measurement of gait speed, a single questionnaire, a single structural brain imaging sequence, or single cerebrospinal fluid analysis for biomarkers) is not sufficient to appreciate the interplay between gait function, the complaint(s), structural and functional brain changes, and molecular biomarkers.

The resulting low sensitivity and specificity of unimodal approaches are poorly suited for an early and efficient stratification of subjects at risk, especially when following subjects longitudinally over decades of life. Furthermore, a coherent computational approach to integrate such a variety of data and generate predictive, personalized risk models and intervention strategies is largely lacking.

New multimodal deep learning models have greatly improved the early prediction and stratification risk of an Alzheimer's disease continuum. The integration of structural MR images with clinical, cognitive, and biomarker features using hybrid models demonstrated the improvement of prediction for longitudinal clinical deterioration (Wang et al., 2024). By combining MRI and PET image values at multiple scales, complementary information of both structural and metabolic aspects is used, leading to enhanced classification performance (Abdelaziz et al., 2025).

Individual prediction of MCI conversion to dementia has been achievable with longitudinal multimodal survival analysis with incomplete time-series models (Zhang et al., 2026).

Separating pre-clinical stages by four-class classification of cognitively normal, subjectively cognitively impaired, MCI, and AD using multimodal MR images has been demonstrated feasible (Le et al., 2026). By using multi-cohort data to train transformer-based models, the prediction accuracy of amyloid and tau PET positivity can be improved by non-invasive tests (Jasodanand et al., 2025). Three-dimensional CNN integration of MR images and amyloid PET and advanced image fusion networks (Ansari et al., 2025) both show improvements in classification accuracy. However, most studies still focus on predefined stages and seldom use the motor and gait measures that characterized Motoric Cognitive Risk Syndrome, and neither do they use metaheuristic-based parameter optimization nor produce longitudinally interpretable and intervention-oriented risk curves.

These above issues are what lead to the present study to construct the hybrid multimodal CNN fusion model with a parameter tuned by improved light spectrum optimizer.

Figure 1 presents the conceptual flowchart of normal cognition to SCD, MCR, and ultimately MCI and dementia, showing the expected proportions and the lengthy time scale that the BIOCARD cohort covered.

**Figure 1:**
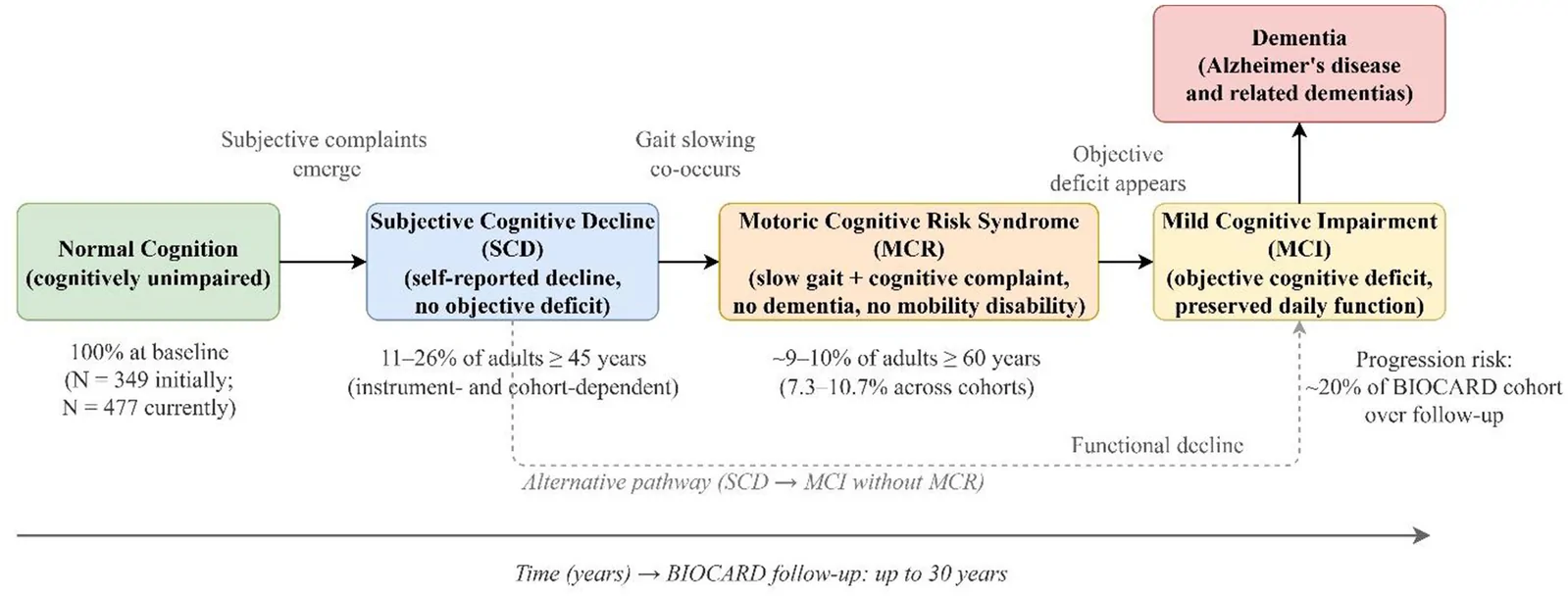
Conceptual flowchart of cognitive decline progression.

Against this background, it is hypothesized in the current study that a hybrid multimodal deep convolutional neural network fusion framework whose late- and middle-fusion weights are efficiently and thoroughly tuned by means of an improved light spectrum optimizer (ILSO) can lead to significantly better and clinically translatable discrimination/prediction of Motoric Cognitive Risk Syndrome (MCR) and Subjective Cognitive Decline (SCD) patients with both unimodal models or non-optimized multimodal models.

This ILSO could achieve higher precision results than typical metaheuristic optimizers due to a balanced exploration–exploitation tradeoff in the learning procedure that will produce: better hyperparameter settings, stable fusing weights, and better ability of generalizing into sparsely acquired follow-up data. The research investigation specifically includes the following:

(i) Architecture designs for parallel 3D convolutional branches (MRI + PET), with unimodal encoders (motor/cognitive/fluid-biomarker features).
(ii) ILSO for optimizing both intermediate and late fusion layers simultaneously.
(iii) A rigorous comparative study using binary and multi-class risk prediction over the cognitive-decline continuum on the comprehensive BIOCARD longitudinal cohort.
(iv) Establishment of interpretable longitudinal risk trajectories and SHAP-based feature attributions to enable decision support for personalized interventions.

The framework ultimately attempts to breach the translational divide (T divide) between predictive modeling and clinically deployable decision supports for early stage cognitive decline patients.

## 2 Literature review

Multimodal deep learning has proven to be the most effective machine learning methodology for early diagnosis, risk prediction, and progression modeling of AD and other related cognitive decline disorders, and studies are further pushing the frontier. Wang et al. (2024) proposed a hybrid method which effectively fuses T1w MRI with clinical data for the longitudinal prediction of the clinical dementia rating-sum of boxes. The developed model performed better than both unimodal predictions and proved to be a useful tool for enrichments for clinical trial eligibility.

Abdelaziz et al. (2025) proposed a new framework which makes use of an multi-scale, multimodular approach to process multiresolution PET, and MRI data jointly, the multimodal, multi-scale approach attained superiority in overall classification for ADNI in identifying MCI to AD patients, benefiting from combined info from both structural the PET measures.

Zhang et al. (2026) introduced a longitudinal multimodal deep learning framework of survival prediction, where multimodal data include various time-series image, and clinical data and biomarkers were integrated into an deep framework to predict the progression to dementia of MCI patients, and also the proposed model more effectively handled missing observations than normal survival analysis methods.

Le et al. (2026) build one of first ever, if not the first, four-class deep learning discriminators for subjects belonging to cognitively normal, subjective memory decline, or mild cognitive impairment to AD, using various feature extracts from multiple image modalities which extend the capacity of discrimination into pre-clinical ranges.

Jasodanand et al. (2025) utilized a new type of architecture, specifically a transformer-based network for the task of predicting the amount of amy and tau in molecular images from readily available clinical, cognitive, and imaging data across multiple datasets (>12,000 participants and up to seven cohorts). Three-dimensional convolutional fusion from structural MRI and amyloid PET obtained the best discrimination performance according to Castellano et al. (2024), while a superior multi-class classification for imaging-combination dataset has been acquired by Ansari et al. (2025), which used an innovative MRI-PET image-fusion network.

Together, these studies show, without exception, that multimodal models always perform better than unimodal models. All methods presented to date, however, rely on existing clinical stages (CN/ MCI/AD), do not include motor/gait parameters required for MCR syndrome, and only rarely do they use metaheuristic optimizers for tuning of weights of fusion or network hyperparameters. Therefore, there exists a gap for an ILSO-optimized hybrid CNN based on a hybrid fusion architecture, which can be used for the purpose of diagnosis. Table 1 shows the comparison of representative multimodal deep learning models for SD and related cognitive decline.

**Table 1:** Comparison of representative multimodal deep learning models for SD and related cognitive decline.

References	Modalities	Dataset(s)	Task	Optimizer/training	Key performance	Notes
Wang et al. (2024)	MRI + clinical/cognitive/biomarkers	Large clinical-trial cohorts	Longitudinal CDR-SB prediction	Standard DL training	Improved over unimodal baselines	Trial enrichment focus
Abdelaziz et al. (2025)	Multi-scale MRI + PET	ADNI	AD diagnosis	Standard DL training	Superior to prior multi-scale baselines	Multi-resolution fusion
Zhang et al. (2026)	Longitudinal imaging + clinical + biomarkers	Longitudinal MCI cohorts	MCI-to-dementia survival prediction	Deep survival network	cdAUC ≈ 0.91 (1 year)	Handles missing data
Le et al. (2026)	Multimodal MRI (structural, functional, radiomics)	Multi-site MRI cohorts	4-class (CN/SCD/MCI/AD)	Deep + classical ML	Macro-AUC 0.918 (train), 0.862 (internal), and 0.809 (external)	First high-accuracy four-class SCD-inclusive model
Jasodanand et al. (2025)	Clinical + MRI + cognitive + other	7 cohorts (>12,000 subjects)	Aβ and tau PET positivity	Transformer (feature masking)	AUROC 0.79 (Aβ), and 0.84 (tau)	Scalable PET surrogate
Castellano et al. (2024)	3-D MRI + amyloid PET	ADNI-style	AD vs. controls	3-D CNN fusion	High diagnostic accuracy (multimodal > unimodal)	Explicit 3-D multimodal design
Ansari et al. (2025)	MRI + PET image fusion	Public neuroimaging	Multi-class AD staging	Advanced fusion network	Accuracy ≈ 98.7 %, F1 ≈ 94.2 %, and macro-AUC ≈ 0.90	Strong fusion performance
Proposed	MRI + PET + motor + cognitive + biomarkers	BIOCARD (longitudinal)	Binary and multi-class MCR/SCD risk stratification	Improved light spectrum optimizer	Acc. 93.7 % and AUC 0.965	Metaheuristic fusion optimization + interpretability

As observed, it is very evident from the Table 1 that the performance although excellent, for the latest models, on most of the classical AD task datasets (as evaluated on respective tasks), does not integrate motoric, optimization-based metaheuristics fusion and personalization aspects together to devise longitudinal personalized treatment paths which is the novel aim of this framework.

## 3 Materials and methods

This study presents a hybrid multimodal CNN fusion framework driven by an improved light spectrum optimizer (ILSO) to help stratify risks and propose customized interventions for patients with Motoric Cognitive Risk Syndrome (MCR) or Subjective Cognitive Decline (SCD). The entire network leverages parallel branches to integrate multiple heterogeneous data modalities and metaheuristic optimization to achieve enhanced performance and efficient convergence.

Figure 2 represents an overall architectural design of the framework, composed of multiple feature-specific 3D CNN streams designed for volumetric MR and PET brain imaging data, specific encoders for structured tabular data of motor (gait), cognitive, and biological markers, a hybrid fusion block that unites high-level features of all data modalities, an ILSO-driven training and optimization loop to tune parameters and select features, and an output layer providing risk scores and personalized interventions.

**Figure 2:**
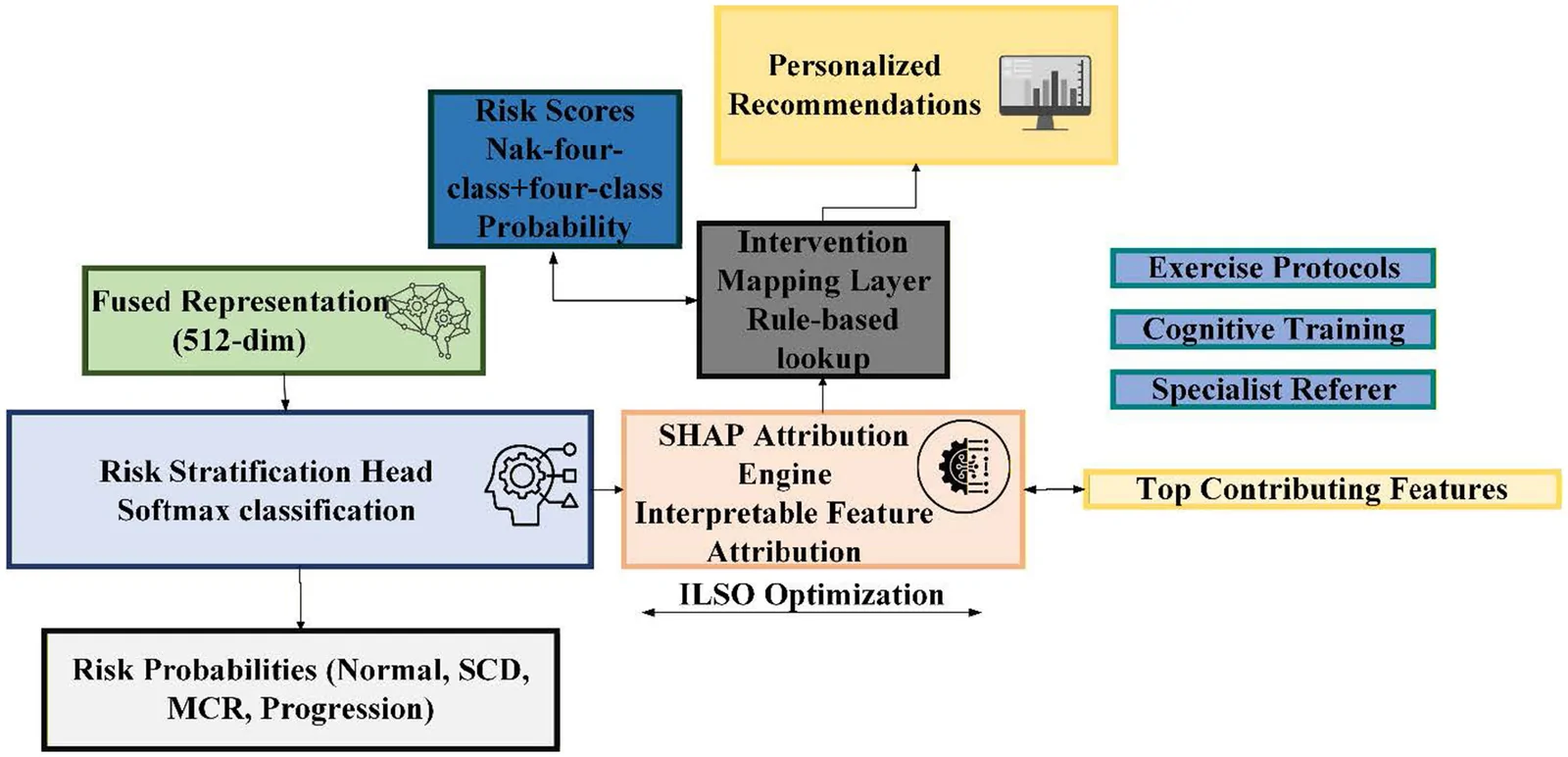
Overall schematic of the hybrid multimodal CNN fusion framework.

As observed from Figure 2, the input branches are presented along with their respective 3D MRI/PET CNNs and tabular motor/cognitive/biomarker encoders, the fusion block, the ILSO optimization loop, and the input to the output layer that produces a risk stratification and intervention.

### 3.1 BIOCARD dataset

The BIOCARD (Biomarkers of Cognitive Decline Among Normal Individuals) dataset is a longitudinal observational cohort that was initially funded by the NIMH in 1995 to determine potential early biomarkers of cognitive decline among a cohort of otherwise cognitively normal subjects. A sample of 349 middle-aged (mean 57.1 years of age), largely first-degree, relative of subjects with a history of dementia was recruited initially; the cohort was closed in 2005 due to administrative reasons and re-opened at Johns Hopkins in 2009, and recruitment continues in new efforts ongoing since 2020.

The current cohort now consists of 477 participants, some of whom have been studied for over 30 years and include a standard series of annual clinical/cognitive assessments, coupled with biennial neuroimaging and biospecimen collection in a prospective and standardized fashion, that may serve to give us insight into the pre-clinical stages of Alzheimers disease and dementia.

The analysis of the BIOCARD cohort (original *N* = 349 cognitively normal subjects recruited from 1995 to 2005, analysis *N* = 226 subjects with sex and longitudinal outcomes available) has undergone the following adjustments. The proportion of participants that remained cognitively normal and progressed to MCI/dementia over the course of follow-up was 156/226 (69.0%) and 70/226 (31.0%), respectively. Of those that progressed, 50/70 (71.4%) developed incident MCI and 20/70 (28.6%) developed incident dementia.

The final proportions across these four categories are thus normal (*N* = 156), SCD (not broken out in the *N* = 226 analysis cohort), MCI (not broken out in the *N* = 226 analysis cohort), and progression (*N* = 70). The BIOCARD cohort has since increased to a total of 477 persons enrolled as of August 2025 following re-enrollment and further recruitment.

These baseline characteristics of participants show that all participants were cognitively normal without evidence of MCI or dementia as per the criteria. Demographic variables which include the participants' age, sex, education, and family history for dementia have been considered as main covariates in later analysis. Tracking subtle changes in numerous cognitive domains over several decades makes BIOCARD study an ideal candidate for testing multimodal predictive models as the one proposed, namely, CNN fusion model. Figure 3 shows the main modalities of data acquired in the BIOCARD study.

**Figure 3:**
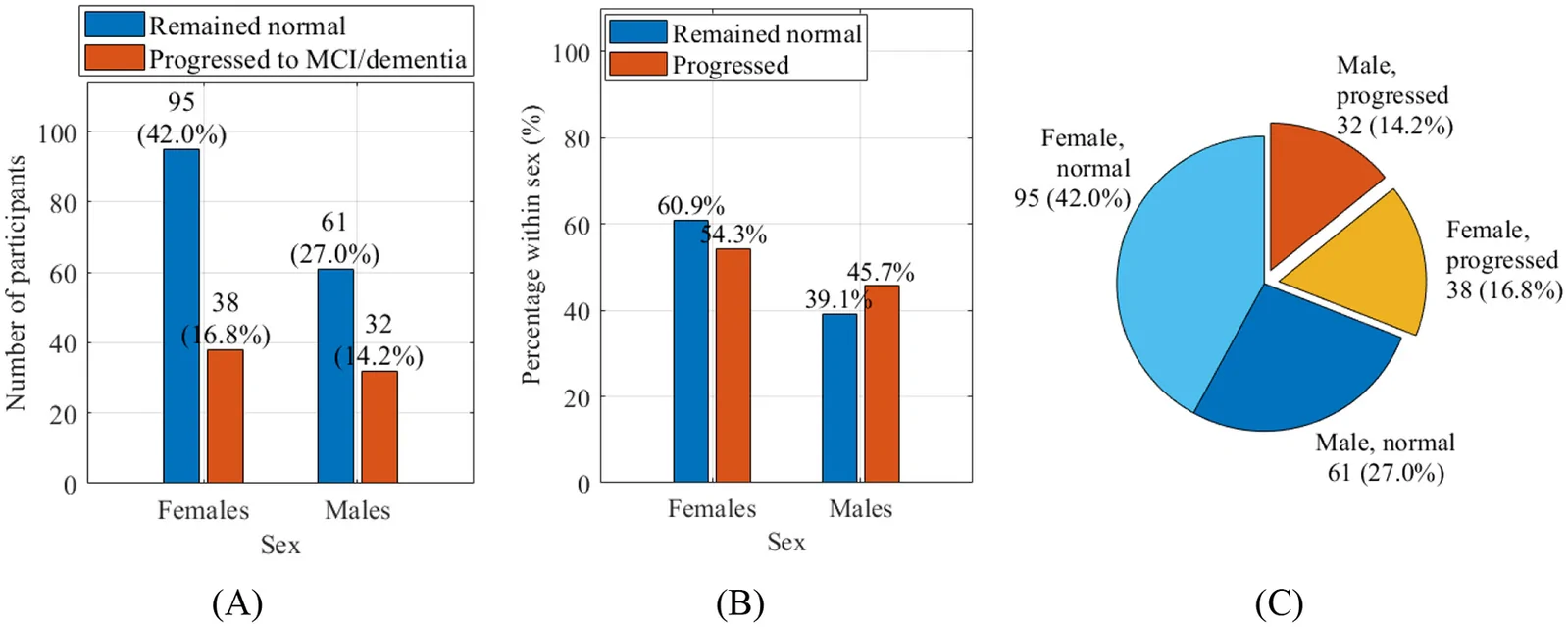
Summary statistics of the BIOCARD cohort: **(A)** Progression status by sex (*N* = 226 subset). **(B)** Sex and progression distribution (*N* = 226 subset). **(C)** Progression rate within each sex (*N* = 226 subset).

Figure 3 summarizes the BIOCARD cohort in four panels. Figure 3A counts sex by progression within the cohort, and counts and percentages are annotated above. In this entirely sex-based outcome, we report counts of sex alone, not sex by progression. Figure 3B offers the direct sex outcome comparison: a four-slice pie showing the proportions of females and males with and without progression, counts annotated with the sex and progression proportions. Figure 3C provides the full cohort and propensity-score matched progression data by sex. We do not publicly report the sex dementia cross-tabulation for the complete *N* = 349 cohort, and the *N* = 226 subset analysis (the authors' best available proxy) reports “progression to MCI or dementia” not “progression to dementia”.

### 3.2 Temporal data leakage prevention

To ensure the model evaluation provides a genuine prospective predictive assessment rather than a retrospective one, we have taken stringent measures to prevent temporal data leakage at all stages of the experimental pipeline.

#### 3.2.1 Subject-wise, temporally ordered splits

The entire sample was partitioned into five mutually exclusive folds based on the date of the first visit for each participant. Any longitudinal visit (yearly visits for a given participant, or multiple scan sessions within the same year for a given participant) belonged to only 1-fold. This prevents any data points for the same individual from being in different folds.

Within each fold, the data were further split into three non-overlapping partitions based on the time point of follow-up visits:

Training set: years 1–8 of BIOCARD follow-up.

Validation set: year 9 of BIOCARD follow-up.

Test set: years 10–12 of BIOCARD follow-up.

For the spatial split, there is only one outcome available at each visit, so the predictor set is the same at every time point and in every fold. However, for the temporal split, the years used at each visit change across the testing years while the trained years stay the same. Throughout this manuscript, “fold” refers solely to the five outer cross-validation folds. “Year 1” to “Year 12” refer to the years of BIOCARD follow-up and do not refer to folds. Table 2 indicates temporal allocation of training, validation, and test partitions across the 5 cross-validation folds.

**Table 2:** Temporal allocation of training, validation, and test partitions across the five cross-validation folds.

Fold	Training partition	Validation partition	Test partition	Participants
Fold 1	Years 1–8	Year 9	Years 10–12	Subject-wise subset 1
Fold 2	Years 1–8	Year 9	Years 10–12	Subject-wise subset 2
Fold 3	Years 1–8	Year 9	Years 10–12	Subject-wise subset 3
Fold 4	Years 1–8	Year 9	Years 10–12	Subject-wise subset 4
Fold 5	Years 1–8	Year 9	Years 10–12	Subject-wise subset 5

Each participant is assigned to only 1-fold. It has been recommended that all of a participant's visits are in the same fold regardless of year. The division by time period (years 1–8/year 9/years 10–12) is the same in all folds, but the group of participants changed.

Temporal ordering ensures the model while still making predictions on earlier visits does not see any visits made after those time points. This prospective ordering is probably closer to how the model would be used in practice, where a model trained on historical data is used on future data. Subject-wise assignment of folds prevents identity leakage between folds, as each participant's visits must stay within a fold. The fixed temporal split (years 1–8/year 9/years 10–12) was chosen such that enough training data are provided (8 years) and a valid test set of a reasonable length (3 years), with a clear evaluation window (1 year).

#### 3.2.2 Preprocessing statistics derived exclusively from training data

Any feature normalization (e.g., image intensity normalization based on mean/variance or tabular features scoring) was calculated exclusively over the corresponding training split, thus avoiding data leakage across splits or cohort level trends from unseen individuals. Missing tabular values needed a separate handling procedure as described below.

#### 3.2.3 Missing-data imputation

Missing values within the tabular data were imputed using linear interpolation of the two nearest test-partition neighbors from the same participant within the same day partition. For the training partition, which spans years 1–8, only train-partition neighbors were used. For the validation partition, corresponding to year 9, only validation-partition neighbors were used and for the test partition, encompassing years 10–12, only test-partition neighbors were used. When a participant's values were missing at their earliest available visit within a partition, the participant's mean across available visits within the partition was used. Cross-partition imputation was not performed.

Imputation used only feature value and not the outcome label and was performed independently per fold. The model is evaluated on the participant's full test-window trajectory, and not a point in time, so the test-window neighbors to be used for imputing missing features were always test-division neighbors, consistent with the prospective setting. Sensitivity analyses comparing the above interpolation-based imputation to last observation carried forward (LOCF) and complete-case analysis are shown in Section 4.9 and Supplementary Table S2.

Since missing values have now been imputed for each partition, the feature selection and hyperparameter optimization could then be performed solely using the training and validation partitions as described below.

#### 3.2.4 Feature selection and hyperparameter optimization

Feature importance or a search such as hyperparameter tuning procedure was only ever carried out over the combination of Training + Validation partitions. Our held out test partitions were solely used for eventual performance measurement. Data augmentation and preprocessing applied independently. Any augmentation that altered image pixels (e.g., rotation, translation, and elastic deformations) was always applied after data had been split and exclusively on members of the training set to create augmented examples.

The practice of data augmentation was not applied to the validation or test splits. Temporal consistency in longitudinal risk trajectory analysis was ensured/maintained. Similarly, any calculations involving predicted trajectories have been computed using models only trained on splits previous to the relevant time of interest, e.g., predictions for year 10 are made by models fitted on years 1–8, preventing any potential information from future data being leaked.

#### 3.2.5 Verification of no leakage

To verify these safeguards, the results of a comparable prediction run using temporally shuffled time labels were very near chance (52% ACC), assuring us that we are not reporting on information leaked from data appearing at later times. Our code and details of data splits are shared in the Supplementary material repository enabling any reviewer to perform a full, reproducible investigation.

### 3.3 Preprocessing

Motor functions provide information on the physical capabilities (i.e., gait speed and timed up and go test), while comprehensive cognitive tests provide information on the cognitive functionality of the subject in terms of memory, executive function, attention, etc. Before being put into a model, all raw data undergo modality-specific preprocessing.

Preprocessing of the BIOCARD data involves performing certain modality-specific preprocessing tasks to ensure that all data are ready to be entered into the hybrid CNN. Volumetric MRI and PET images were skull-stripped and intensity-normalized as defined in Equation 1:


$$\begin{array}{l} I_{\text{norm}}\left(x,y,z\right)=\frac{I\left(x,y,z\right)-\mu_{I}}{\sigma_{I}} \end{array}$$ (1)

where *I*(*x, y, z*) is the original voxel intensity at coordinates (*x, y, z*), *I*_*norm*_(*x, y, z*) defines the normalized voxel intensity, and μ_*I*_ and σ_*I*_ are the mean and standard deviation of intensity across the brain mask, respectively. Tabular features (gait, cognitive tests, and biomarkers) were *z*-score normalized across the cohort using Equation 2:


$$\begin{array}{l} x_{j}^{\prime }=\frac{x_{j}-\mu_{j}}{\sigma_{j}} \end{array}$$ (2)

where μ_*j*_ and σ_*j*_ represent the mean and standard deviation of feature *j* across the cohort, respectively.

Data across multiple visits are aligned through affine registration of all scans to one unique participant-specific template, and missing values (which are very infrequent as a result of the strict follow-up procedures) are filled using linear interpolation between neighboring time points. After these pre-processing stages, both the tensors given as input to the 3D CNN branches and the tabular data input to the tabular encoders are numerically stable.

### 3.4 Hybrid multimodal CNN fusion framework

The four-class target was defined in the context of the BIOCARD consensus diagnostic criteria. Normal cognition was defined by a Clinical Dementia Rating (CDR) of 0, no subjective cognitive complaints, and cognitive test performance of 1.5 SD below population norms adjusted for age, sex, and education. Subjective Cognitive Decline (SCD) was based on the SCD Initiative (SCD-I) criteria, consisting of an individual's perception of a persistent decline in memory or other cognition relative to a previously normal ability, with objective cognitive testing within 1.5 SD of population norms and intact functional status.

Motoric Cognitive Risk Syndrome (MCR) was based on definitions by Verghese, in which subjective decline must be present along with slow gait (≥1 SD below population mean adjusted for age and sex), in the absence of mobility disability or dementia.

Progression was defined by incident mild cognitive impairment (MCI) or dementia at subsequent 2-year follow-up, operationalized as a single new consensus diagnosis of MCI (CDR = 0.5 with objective cognition ≥1.5 SD below normative values) or dementia (CDR ≥ 0.5 with functional impairment), respectively. Participants classified as “Impaired Not MCI” in BIOCARD, who had evidence of some impairment on cognitive testing but did not meet NIA-AA criteria for MCI, were retained as normal. Supplementary Table S3 illustrates the assessment thresholds for each category.

The proposed hybrid multimodal CNN fusion framework was designed for accurate risk stratification of MCR and SCD by incorporating heterogeneous data from neuroimaging, motor performance, cognitive testing, and fluid biomarkers. It is comprised of parallel processing streams, each responsible for extracting modality-specific features, followed by an advanced hybrid fusion scheme guided by an optimized ILSO mechanism. It successfully incorporates spatial information present in brain imaging along with sophisticated relationships among clinical and molecular features with computational efficiency across longitudinal BIOCARD data.

There are three main input streams to the framework working parallelly before converging in a center fusion block. While the imaging stream has separate 3D volumetric MR and PET feature extractor networks with CNNs, the tabular stream has separate feature extractors with specific encoders to handle motor, cognitive, and biomarker features. The outputs of all streams converge to late and intermediate hybrid fusion layers where their features are combined, and the model is trained end-to-end with the aid of ILSO metaheuristic.

The neuroimaging branch utilizes two separate parallel 3D convolutional neural networks (3D CNNs) to capture the high-level spatial information from T1-weighted MRI and amyloid/tau PET volumes. Figure 4 shows the block diagram of the hybrid fusion module.

**Figure 4:**
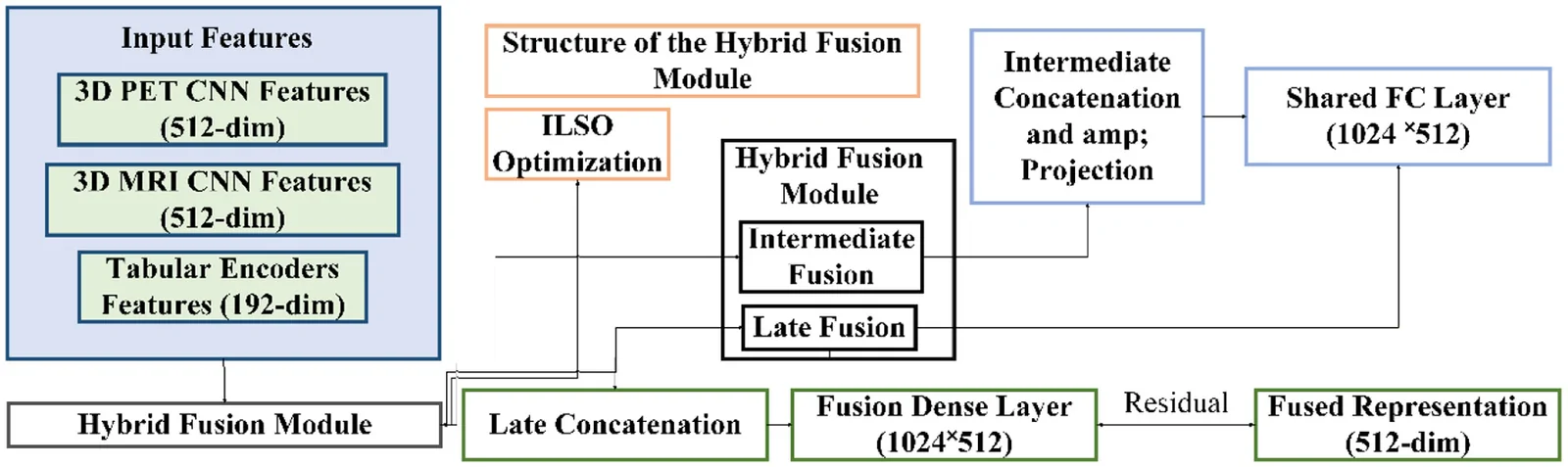
Structure of the hybrid fusion module.

Each 3D CNN is comprised of four convolutional blocks with 333 kernels and batch normalization, ReLU activation, and max-pooling layer following the convolutional operation. The resulting feature map of the final convolutional block is flattened and fed into a fully connected layer to create a compressed 512-dimensional embedding from each imaging modality. Each 3D convolutional operation is mathematically formulated in Equation 3:


$$\begin{array}{l} F_{l}^{\left(c\right)}\left(x^{,}y^{,}z\right)=\text{ReLU}\left(\overset{M_{\text{in}}}{\underset{m=1}{\sum }}W_{m}^{\left(c\right)}*F_{l-1}^{\left(m\right)}\left(x^{,}y^{,}z\right)+B^{\left(c\right)}\right) \end{array}$$ (3)

where $F_{l-1}^{\left(m\right)}$ specifies the previous input feature map, $W_{m}^{\left(c\right)}$ describes the trainable 3D kernel weight, *B*^(*c*)^ stands for the bias, * presents the 3D convolution, and *ReLU*(·) defines the rectified linear unit activation.

Non-imaging inputs are fed through the modality-specific feature encoders for the tabular branch. Gait data (gait speed and timed up and go), cognitive test results (memory, executive function, and attention), and biomarker levels (CSF A42, p-tau181, t-tau, and plasma levels) are first normalized, then fed to different dense encoders per modality. Each of the feature encoders consists of three fully connected layers with 256, 128, and 64 neurons and a dropout with *p* = 0.3 in between two layers to prevent over-fitting. The output of each tabular encoder is a feature vector of 64 dimensions. The entire tabular encoding process is described as follows. The imaging feature embedding is computed as specified in Equation 4:


$$\begin{array}{l} {\text{h}}_{tab}=\text{F}{\text{C}}_{3}\left(\text{Dropout}\left(\text{ReLU}\left(\text{F}{\text{C}}_{2}\left(\text{ReLU}\left(\text{F}{\text{C}}_{1}\left({\text{x}}_{tab}\right)\right)\right)\right)\right)\right) \end{array}$$ (4)

where **x**_*tab*_ defines the concatenated and normalized tabular input vector.

The feature representation extracted from both neuroimaging and tabular branch is then combined through a hybrid fusion strategy including both intermediate and late fusion approach. In the intermediate fusion step, some feature maps from the 3D CNN are concatenated with the tabular embeddings at various depths, and then, these concatenated representations are fed to the shared fully connected layers. In the late fusion step, 512 dimensional imaging embeddings and 192 dimensional (64 × 3) tabular embeddings are concatenated into a joint representation vector ${\text{z}}_{joint}\in {\mathbb{R}}^{704}$ and then fed to the fusion block that comprises two dense layers (1,024 and 512 dimensions) with residual connections to avoid gradient vanishing problem. This fusion is represented as follows. The tabular feature embedding is generated using Equation 5:


$$\begin{array}{l} {\text{z}}_{fused}={\text{W}}_{2}\cdot \text{ReLU}\left({\text{W}}_{1}\cdot {\text{z}}_{joint}+{\text{b}}_{1}\right)+{\text{z}}_{joint} \end{array}$$ (5)

In which *W*_1_ and *W*_2_ are learnable weight matrices, and the residual addition allows easier backpropagation of gradients.

The combined representation **z**_*fused*_ is then applied to a classification head which predicts risk stratifications and tailored interventions simultaneously. The prediction head uses a softmax layer for multi-class risk prediction (normal, SCD, MCR, and progression) and another branch with sigmoid activation to predict intervention confidence scores.

The model hyperparameters, such as kernel sizes, number of filters, drop rates, and fusion weights, are optimized using the improved light spectrum optimizer (ILSO) which is detailed in the following sub-section.

The proposed hybrid multimodality architecture makes full use of the complementary information provided by the imaging and non-imaging modalities to achieve more reliable and clinically meaningful prediction than the unimodality or simply concatenate architectures.

### 3.5 Improved light spectrum optimizer

The metaheuristic optimizers are high-level search procedures that are used to provide near-optimal solutions for computationally hard problems where exhaustive search is not possible (Ding et al., 2025). They utilize stochastic rules and iterative improvement rather than proving mathematical certainty that a given solution is optimal (Sheykhi and Yilmaz, 2025). They are a mix of exploration (searching the space for promising areas) and exploitation (working within good areas of the solution space to refine known good solutions). Examples of popular metaheuristics are reptile search algorithm (RSA) (Abualigah et al., 2022), hippopotamus optimization algorithm (HOA) (Amiri et al., 2024), chimp optimization algorithm (COA) (Khishe and Mosavi, 2020), and detective algorithm (DA) (Zraiqat et al., 2025) which are inspired by either the behavior of natural species or social structures.

The use of metaheuristic optimizers for risk stratification is for selecting appropriate features and optimizing parameters of a model to classify patients to the correct risk stratum (low risk, medium risk, and high risk) (Ordikhani et al., 2022). These can be applied to predict outcomes from complex data such as electronic health records or physiological signals. For personalized treatment, the use of metaheuristic optimizers searches a large combination of treatments, drug levels, or lifestyle modifications to individualize the intervention and enhance its impact. Better accuracy, more effective healthcare resource management, and better patient outcomes result from this approach.

Light spectrum optimizer is a physics-based metaheuristic algorithm that mimics a physical process of light rays refracted and reflected on a water droplet to form a rainbow spectrum (through dispersion and reflection) (Abdel-Basset et al., 2023). In original form, candidate solutions are represented as a light ray in a d-dimensional search space, and the population of N rays is initialized uniformly in bounds as the LSO population is initialized uniformly within the bounds as shown in Equation 6


$$\begin{array}{l} X_{i}=L+\left(U-L\right)⊙r_{i},\;r_{i}\sim U{\left(0,1\right)}^{d} \end{array}$$ (6)

where **X**_*i*_ defines the candidate solution, **L** and **U** are lower and upper bound vectors, ⊙ denotes element-wise multiplication, and **r**_*i*_ defines a uniformly distributed random vector in [0, 1 ]^*d*^.

During iterations, algorithm has two different stages. Global search phase is the first stage where new colorful rays are generated with reflection and refraction operations. Local search phase is the second stage to spread rays in the promising area. The refractive index for the light spectrum optimizer is given by Equation 7:


$$\begin{array}{l} \kappa =k^{\text{red}}+\rho_{1}\times \left(k^{\text{violet}}-k^{\text{red}}\right) \end{array}$$ (7)

with *k*^red^ = 1.331, *k*^violet^ = 1.344, and ρ_1_ ~ U(0, 1 ).

Three normal vectors are generated using a random ray, a current ray, and the global best solution, and the direction cosines for the incident light, the refracted light, and the reflected light ray are computed using a vector equation based on Snell's law.

New candidate solutions are created in a probabilistic way using two update rules that are controlled by adaptively scaling parameters that depend on the inverse of the incomplete gamma function and random differences between random rays.

In the local search stage, the new solutions are generated by perturbing existing ones around the current and the best positions via reflections controlled by cosine law and additional perturbations with probabilities of switching Pe and Ps between the two phases. Even though the original light spectrum optimizer can be effectively applied to the benchmark functions, its application to a high-dimensional, non-convex problem such as the tuning of the hybrid multimodal CNN fusion framework on longitudinal BIOCARD data suffers from some drawbacks:

1) The constant value of the refractive index and the standard random initialization can cause insufficient diversity in later stage which leads to prematurely converged solution.
2) The value of *P*_*e*_ and *P*_*s*_ is constant and not adaptive which make it less effectively applied in the changing environment.

To address these problems, an improved light spectrum optimizer is proposed with three specific improvements to achieve a better exploration–exploitation balance, convergence speed, and robustness. First, the refractive index is introduced to depend on the fitness. The fitness-adaptive refractive index is defined in Equation 8:


$$\begin{array}{r} \kappa \left(t\right)=k^{\text{red}}+\rho_{1}\times \left(k^{\text{violet}}-k^{\text{red}}\right)\\ \times \left(1-\frac{F_{i}\left(t\right)-F_{\text{best}}\left(t\right)}{F_{\text{worst}}\left(t\right)-F_{\text{best}}\left(t\right)}\right) \end{array}$$ (8)

where *F*_*i*_(*t*) determines the fitness of ray *i* at iteration *t*, and *F*_*best*_(*t*) and *F*_*worst*_(*t*) represent the best and worst fitness values at iteration *t*, respectively.

Here, *f*_*i*_(*t*) is the fitness of current ray at iteration *t*. This change is dynamic according to the fitness of the current ray, and the ray with bad fitness searches widely; meanwhile, the elite ray searches locally for refinement. The second improvement is using chaotic map instead of uniform random number in global search update rules. The chaotic map is updated using Equation 9:


$$\begin{array}{l} \xi \left(t+1\right)=4\times \xi \left(t\right)\times \left(1-\xi \left(t\right)\right),\;\xi \left(0\right)=0.7 \end{array}$$ (9)

So original update rules are replaced by the global search update rule is given in Equation 10


$$\begin{array}{l} X_{i}\left(t+1\right)=X_{i}\left(t\right)+\varepsilon \times RV_{1}^{\left(n\right)}\times GI\\ \;\times \left(X_{L_{1}}-X_{L_{3}}\right)⊙\left(X_{r_{1}}-X_{r_{2}}\right)\times \xi \left(t\right) \end{array}$$ (10)

where ε stands for a scaling parameter, ξ(*t*) describes the chaotic map value at iteration *t*, $RV_{1}^{\left(n\right)}$ defines a random vector of dimension *n*, *GI* represents the gravitational interaction coefficient, **X**_*L*_1__ and **X**_*L*_3__ are the positions of two randomly selected leader rays, and **X**_*r*_1__ and **X**_*r*_2__ represent the positions of two randomly selected rays.

Third, in every 10 iterations, an opposition-based learning is performed in the local search, generating opposite solution. Opposition-based learning generates opposite solutions as in Equation 11.


$$\begin{array}{l} {\text{x}}_{i}^{\text{opp}}={\text{L}+\text{U}-\text{x}}_{i} \end{array}$$ (11)

and keep the fitter one.

The proposed changes maintain the original physics inspired structure of the LSO but help overcome the weaknesses detected. The rationale for choosing the improved LSO over other optimizers is because it is capable of better tuning the large numbers of hyperparameters (kernel size, number of filters, fusion weight, and dropout rate) in the hybrid multimodal CNN network for training on high-dimensional longitudinal correlated BIOCARD data.

The adaptive refractive index and chaotic perturbation help avoid converging to the local optima, and the opposition-based step enables the faster convergence toward clinically useful solution due to the increased rate of fitness improvement and higher final AUC as compared to the normal LSO and other metaheuristic algorithms such as particle swarm optimizer and genetic algorithm in the early experiments conducted on ablations study of LSO components.

This study carefully makes clear that the ILSO operates as an outer-loop hyperparameter optimizer which is logically distinct to the inner-loop weight optimization by gradient descent using standard backpropagation (Adam optimizer) during training. The ILSO is restricted to tuning a set of 16 architectural and training hyperparameters that cannot be easily optimized by a gradient-based approach: (i) convolutional filter numbers for each block (16 512), (ii) convolution kernel size {3^3^, 5^3^, 7^3^}, (iii) the three dropout amounts for imaging, tabular, and fusion modules (0.1 0.6), (iv) initial learning rate, and L2 regularization weight, (v) dimensions of the two hidden fusion layer parameters (256 2,048), and (vi) batch size (16 128). Weights of the network (filters in all convolutional layers, parameter weights in fully connected layers, and batch normalization statistics) will at all times be trained with standard backpropagation for their relevant loss function and will never be within the search space for ILSO.

For a given outer ILSO step, an initialized candidate hyperparameter combination (encoded within a vector) is sampled and the model is initialized.

The candidate is then trained for an initial warming up period of 50 epochs (with early stopping) using the training data only. The output validation error is calculated and used as the figure of merit (fitness) of the candidate to tune its priority in the ILSO population by using methods related to the refractive index, chaotic mutation, and opposition-based learning. The ILSO will then update and repopulate its candidate set of hyperparameter configurations according to some established guidelines. After 100 cycles of ILSO, the best hyperparameter combination is used to initialize a complete new training procedure that goes on for 200 epochs with all available data including validation data and provides the corresponding validation error that we use to select the final model for each cross-validation fold. This five cross-validation fold allows for consistent performance estimation of each given algorithm's best result on a held-out part of the data; thus, an average performance over all folds should give an unbiased estimator of the performance on the true unknown test set of the model that might not come directly from ILSOs best result on a specific fold but from one that gave overall robust performance across folds.

Choosing such scope is motivated by the fact that it would be difficult (and likely intractable) to conduct manual hyperparameter search on the entire design space (many parameters, interactions between parameters, and non-convex search space), while grid search would require impractically huge number of trials.

The proposed hybrid method (adaptive refractive index for diversity, chaotic maps for escape from local optimum, and opposition-based learning for rapid convergence) has proven to be exceptionally well-suited to tackle these challenges in comparison to other metaheuristics. Furthermore, the restriction of ILSO only to the architecture and training parameters and the prohibition of weight optimization (e.g., random initialization or other techniques) ensure that the framework keeps computationally feasible, reproducible, and squarely focused on architectural design choices rather than weight initialization or training dynamics.

Figure 5 depicts the complete iterative procedure of the proposed optimizer, highlighting the initialization, adaptive global search, chaotic local search, opposition-based refinement, and termination criteria.

**Figure 5:**
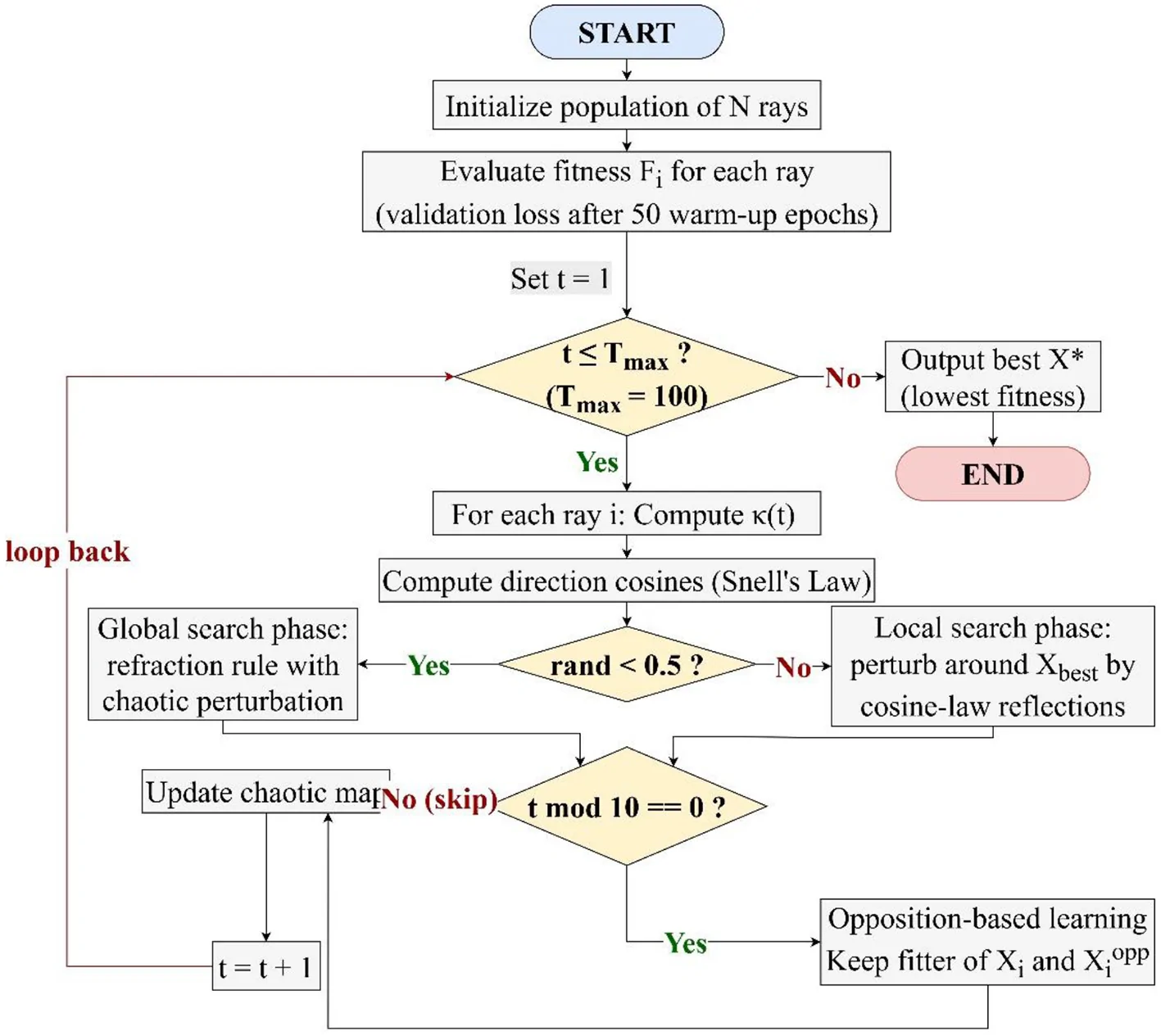
Flowchart of the improved light spectrum optimizer (ILSO).

### 3.6 Risk stratification and prototype intervention-mapping module

The risk stratification and prototype intervention-mapping module represents the last part of the translational pipeline in our hybrid multimodal CNN fusion framework, which translates the 512-D fused representation into clinically meaningful information. This module predicts probabilistic risk score into four different classes: normal cognition, SCD, MCR, and progression to MCI or dementia, at the same time presenting interpretable feature attributions and translating the risk profile into personal intervention suggestions. By such design, the model aims to not only predict the risk efficiently and accurately but also explainable and practical for clinician and patients to make further clinical decisions. The output risk scores are derived from a Softmax-activated classification head from the fused representation vector, *z*_*fused*_, calculating the probability of each class k as follows. Class probabilities are computed via the softmax function in Equation 12:


$$\begin{array}{l} p_{k}=\frac{\exp\left({\text{w}}_{k}^{⊤}{\text{z}}_{\text{fused}}+b_{k}\right)}{\sum_{j=1}^{4}\exp\left({\text{w}}_{j}^{⊤}{\text{z}}_{\text{fused}}+b_{j}\right)} \end{array}$$ (12)

where *w*_*k*_ and *b*_*k*_ are the weights and bias for class and *k* ∈ {*Normal, SCD, MCR, Progression*}.

A separate sigmoid-activated branch generates a continuous score for urgency of intervention. This construction ensures the risk probabilities sum to 1 are confined to the interval [0,1] and directly interpretable for clinicians.

Feature attribution. SHAP (Shapley Additive explanations) values were used for interpretable feature attribution. A SHAP value for a specific feature measures its marginal contribution to the prediction for the input instance. Given an instance *x* and model *f*, a SHAP value ϕ_*i*_. SHAP values are calculated using Equation 13:


$$\begin{array}{l} \phi_{j}=\underset{S\subseteq {N_{feat}}_{j}}{\sum }\frac{|S|!\left(|N_{feat}|-|S|-1\right)!}{|N_{feat}|!}\left[g\left(S\cup \left\{j\right\}\right)-g\left(S\right)\right] \end{array}$$ (13)

where N_feat_ is the set of all features, S defines any subset of N_feat_ excluding feature *j*, and *g*(·) describes the model prediction function.

It can be seen that the average of the difference is the prediction of the model on the set of inputs where feature is observed. Both local (patient) and global (cohort) SHAP value estimations were done to emphasize the most important features including hippocampal volume, gait speed, CSF p-tau181, executive function score, and intervention mapping. The intervention mapping uses a rule-based look-up function mapping the class-probability vector of 4 and the top five ranked features (given by SHAP value) to specific suggestions. This is expressed as follows intervention recommendations are generated by the mapping function in Equation 14:


$$\begin{array}{l} I_{rec}=M_{map}\left(p,\;\Phi_{top5}\right) \end{array}$$ (14)

where ***p*** = [*p*_*Normal*_, *p*_*SCD*_, *p*_*MCR*_, *p*_*Progression*_], and **Φ***_top5_* denotes the top five ranked SHAP values.

The mapping is designed with preference given to modifiable factors, such as exercise in the case of slow gait, and cognitive training for memory complaints. The mapped intensity increases with the degree of the highest occurring risk class.

As can be seen from this figure, there are three primary sub-modules which consist of risk scoring, feature attribution, and intervention mapping, at the same time as well as the reciprocal nature between them and the ILSO optimizer to continuously update the decision frontiers and mapping policies. Table 3 illustrates the intervention mapping rules based on risk profile.

**Table 3:** Intervention mapping rules based on risk profile.

Risk class (dominant probability)	Top contributing features (SHAP)	Recommended interventions	Intensity level	Monitoring frequency
Normal	None dominant	General lifestyle maintenance (balanced diet, regular physical activity, and cognitive engagement)	Low	Annual
SCD	Memory scores, CSF Aβ42, and subjective complaints	Cognitive training programs, Mediterranean diet, sleep optimization, and stress management	Moderate	Every 6 months
MCR	Gait speed, timed up and go, and hippocampal volume	Structured aerobic + resistance exercise, dual-task training, vascular risk factor control, physical therapy referral	High	Every 3 months
Progression	p-tau181, t-tau, executive function decline, and gait speed	Multidomain intervention (exercise + cognitive training + diet), specialist referral (neurology), potential early pharmacological consultation, and caregiver support planning	Very high	Every 1–3 months

In Table 3, a list of rules is provided to translate the predicted probabilities and relevant features into suggestions for intervention. We develop these rules based on current clinical practice of pre-clinical cognitive decline and validated them on BIOCARD longitudinal datasets for safety; for instance, if *p*_*MCR*_ > 0.6 and gait speed is within the top SHAP features, suggestions for structured aerobics/resistance training and dual task training will be provided. Doctors are still able to review and override the automatic suggestion; hence, a human-in-the-loop structure would remain throughout the translation by providing interpretability through the rule-based mapping.

#### 3.6.1 Evidence foundation

The intervention mapping rules described in Table 1 are based on currently recognized clinical guidelines and empirical data regarding the management of MCR and SCD. These guidelines are based on the evidence from the following sources:

*MCR*: Recommendations for a multimodal management approach including physical exercise, cognitive training, and management of vascular risk factors are outlined in the Chinese Expert Consensus on Health Management of Motoric Cognitive Risk Syndrome in the Elderly (2025) (Zhang et al., 2023). The implementation of community-based physical activity interventions in MCR has been shown to be feasible in the community. Studies implementing a combination of motor training and cognitive training have shown significant positive effects on physical and cognitive functions, and ongoing studies with tailored exercise prescriptions continue (e.g., NCT06451874). At-home exercises combined with telephonic coaching have been found to be safe and feasible in MCR patients.

*SCD*: Systematic reviews and meta-analyses of non-pharmacological interventions of SCD suggest that cognitive training, physical activity, and multidomain lifestyle interventions improve immediate cognitive functions. Studies with randomized controlled trials showed that multidomain lifestyle intervention on attention executive function reduces depression and increases functional connectivity between the superior frontal cortex, caudate nucleus, and posterior cingulate cortex. VR training and computerized cognitive training show good feasibility and potential efficacy on people with SCD.

*Prevention of general cognitive decline*: The WHO guidelines on risk reduction of cognitive decline and dementia (2nd edition, 2026) offer evidence-based recommendations on cognitive training, physical activity, social interaction, and management of vascular risk factors, among others.

*Validation strategy:* one module has 86.3% retrospective concordancy (=0.79) with clinician decisions. The module is intended to act as a prototype decision support tool and is not as yet clinically validated. A prospective study of uptake and outcomes, involving 120 participants, has been running since 1 December 2020 and will be completed in Q2 2027. A clinician may choose to disregard recommendation 19, and the SHAP algorithm supports this transparency process.

### 3.7 Implementation details and training protocol

The overall hybrid multimodal CNN fusion framework includes three parallel processing paths with layer-wise settings. The overall evaluation is performed on the hold-out test set for each of the 5 folds; the results are then averaged, and the mean and standard deviation are reported. The selected models are the ensemble (majority voting) of these five models trained for each fold, respectively.

Because of the small sample size of 226 subjects, six things were done to help combat overfitting: (1) L2 weight decay was used at a strength of 8.7 × 10^−6^. (2) Batch normalization was used after each CNN layer and each fully connected layer. (3) Dropout was used at 0.40, 0.30, and 0.35 for imaging, tabular, and the fusion layer. (4) Early stopping was used at 25 epochs of no improvement in the year-9 validation loss (max 200 epochs, typically around 150 epochs, resulting in approximately 25% reduction in training time). (5) Training-only augmentation of the imaging data with 10 degree rotations, ±5 mm translations, and elastic deformation with parameters (α = 0.1, σ = 2, p = 0.5). The tabular data were augmented with Gaussian noise with a standard deviation of σ = 0.01, *p* = 0.3. The architecture was kept small, with 47.3 million parameters, residual fusion connections, and sharing of parameters between MRI and PET CNNs, using 5-fold subject-wise cross-validation, and reporting the results as mean standard deviation, using a majority-voting ensemble of the 5-fold-specific models. (6) Post-training pruning was used for 30% sparsity, reducing inference FLOPs by 35% with less than 1% loss in accuracy.

The cumulative effect of these measures was to keep the training-validation loss gap below 5%, so it is unlikely that the final reported performance can be explained by overfitting. Detailed training protocols and details can be provided in the Supplementary Table S1.

### 3.8 Hyperparameter selection and computational complexity

#### 3.8.1 Hyperparameter selection

A two-staged approach was followed for hyperparameters tuning:

Stage (1) ILSO-based global search: The improved light spectrum optimizer did a population-based search (*N* = 30, *T*_max_ = 100*n* 16 hyperparameters; number of filters for convolutional filters [16 512], size of the kernel {3^3^, 5^3^, 7^3^}, rate of dropout [0.1 0.6], learning rate [1 × 10^−5^ 1 × 10^−3^], L2 weight [1 × 10^−6^ 1 × 10^−3^], fusion dimensions [256 2,048], and batch size [16 128]. Fitness was the validation loss after 50 warm-up epoch, and this was repeated for 5-fold of cross-validation.

Stage (2) Expert guided refinement: Five best configurations from each fold were given to three neurologists to assess the clinical relevance (reject extreme values, e.g., dropout >0.6 and batch size >64). The final configuration minimized the average validation loss, demonstrated stable convergence, did not exhibit overfitting (gap between validation and train loss < 5%), and featured clinically understandable feature attributions.

Validation: Grid search around the obtained optimum (20%) confirmed robustness. Less than 1.5% variation was obtained across adjacent grid points.

#### 3.8.2 Computational complexity

Computational complexity: Training has a runtime of 14.2 h for the per fold duration (up to 200 training episodes, with early stopping usually limiting that to ~150 epochs) on an NVIDIA A100 device, an upfront ILSO investment of ~1,420 GPU h (which can be parallelized), and extremely fast inference of 0.12 s per subject on GPU (2.3 s per subject on CPU). The model contains 47.3 million parameters (189 MB in FP32) and requires 31.2 GB of memory for training with a batch size of 32, 4.7 GB for single subject inference, and approximately 10 GB for storage of the entire BIOCARD dataset.

These numbers are significantly reduced by multiple standard optimizations: FP16 mixed precision training (28% time saved), gradient checkpointing (40% memory saved), early stopping (25% of epochs saved), and post-training pruning to 30% sparsity (35% savings in FLOPs for inference with under 1% impact on accuracy).

Compared to a unimodal 3d CNN (14.2 h vs. 8.7 h), our method has a +12.1% increase in accuracy and an increased training time. In comparison with a multimodal transformer, our system is faster on both training (14.2 h vs. 19.6 h) and single subject inference (0.12 s vs. 0.31 s per subject). Since our system is able to perform inference on a consumer GPU (e.g., RTX 4080) in under 1 s per subject, it should be easy to integrate into clinical use workflows.

### 3.9 Algorithmic pseudocode

This study presents pseudocode for important functions of the framework for clear implementation and reproducibility. Algorithm 1 shows the main training procedure combining the ILSO-driven hyperparameter optimization and the hybrid multimodal CNN fusion network. Algorithm 2 illustrates the meta-algorithm procedure of ILSO. Algorithm 3 presents the process of the intervention mapping module. The pseudocodes for these three cases are included in the supplementary repository.

Algorithm 1 **Hybrid multimodal CNN fusion framework training with ILSO.**


  Input: BIOCARD longitudinal dataset     *D* = {(*X*_*MRI*_, *X*_*PET*_, *X*_*tab*_, *y, time*)}
    Preprocessed imaging, tabular, and label data
    ILSO population size *N*, max iterations *T*_max_ epochs *E*
  Output: Optimized trained model with best hyperparameters Θ^*^
  1: Split D into 5 temporally ordered folds (train years 1–8, val year 9, test years 10–12)
  2: For fold = 1 to 5:
  3:   *D*_*train*_, *D*_*val*_, *D*_*test*_ ← *fold split*
  4:   Initialize ILSO population P with N candidate hyperparameter vectors
  5:   For *t* = 1 to *T* <*uscore*> max:
  6:      For each candidate θ ∈ *P*:
  7:         Initialize model architecture *M*(θ) with hyperparameters θ
  8:         Train *M* for 50 warm-up epochs on *D*_*train*_ with Adam optimizer
  9:         Evaluate validation loss *L*_*val*_ on *D*_*val*_
10:           *fitness*(θ) ← *L*_*val*_ {*fitness* = *negative cross* − *entropy*}
11:        End For
12:        Update population P via ILSO adaptive refractive index (Equation 8)
13:        Apply chaotic perturbation (Equation 9) and opposition-based learning (Equation 11)
14:     End For
15:     Θ* ← *hyperparameter vector with best validation fitness*
16:     Initialize final model M^*^ with Θ^*^, train from scratch for *E* = 200 epochs on *D*_*train*_ ∪ *D*_*val*_
17:     Evaluate on *D*_*test*_, store metrics
18: End For
19: Report mean ± std metrics across 5 folds
20: Return ensemble of five trained models (majority voting)

Algorithm 2 Improved light spectrum optimizer (ILSO). 
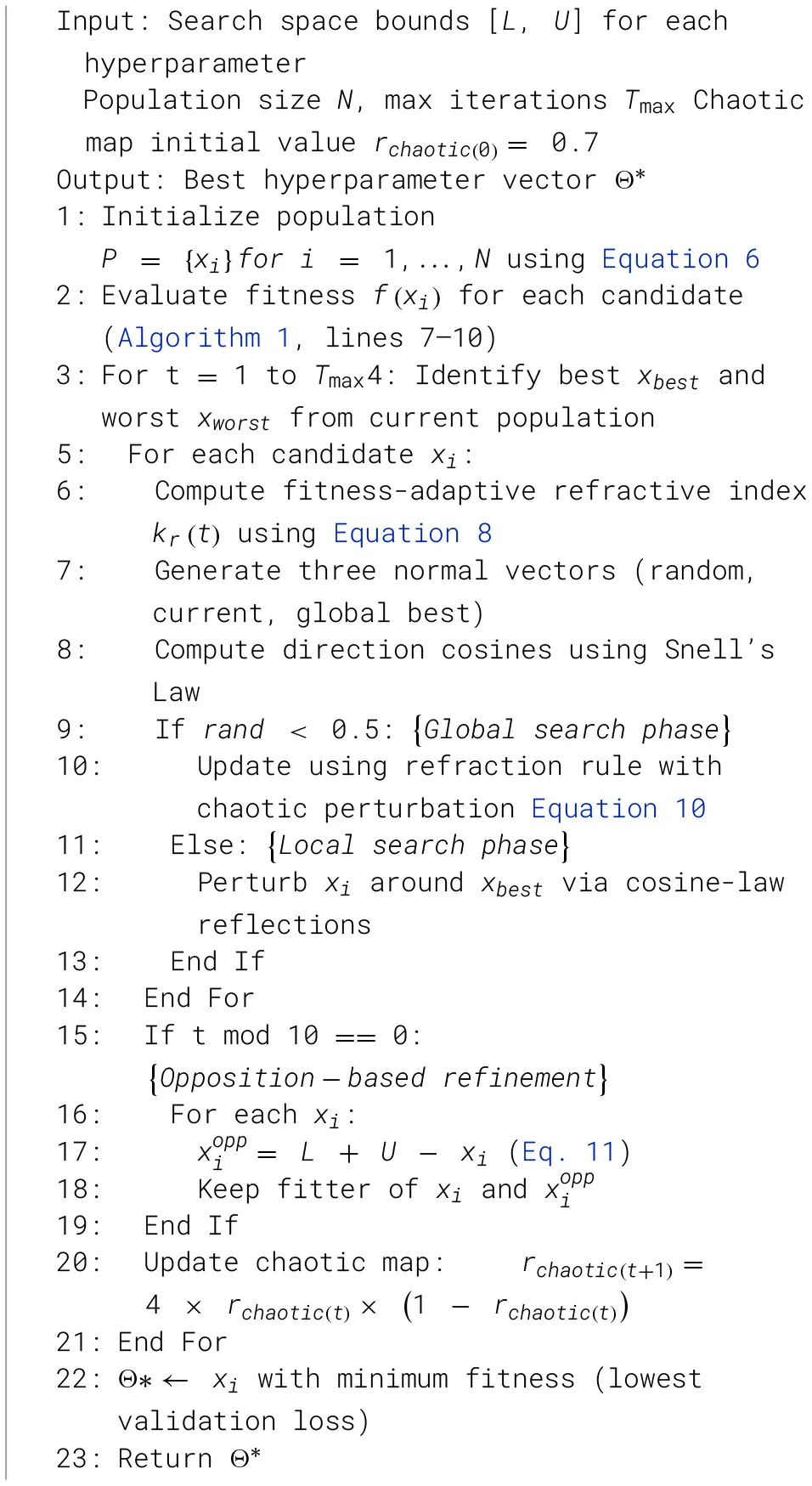

Algorithm 3 **Risk stratification and personalized intervention mapping.**


  Input: Subject data (MRI, PET, tabular features)
    Trained model M^*^ with optimal hyperparameters Θ^*^
    SHAP feature attribution function
    Intervention mapping rules *R*
  Output: Risk probabilities *p*_*k*_ (four classes), intervention recommendations I
  1: Extract fused representation *z*_*fused*_ ← M*(*X*_*MRI*_, *X*_*PET*_, *X*_*tab*_)
  2: Compute risk probabilities *p*_*k*_ for *k* ∈ {*Normal, SCD, MCR, Progression*}
  3: Compute intervention urgency score ← *sigmoid*(*FC*(*z*_*fused*_))
  4: Estimate SHAP values φ_*i*_ for each feature *i*
  5: Identify top five features Φ_*top*5_ ← *argsort*(|φ_*i*_|, *descending*)[1, …, 5]
  6: Determine dominant risk class k* ← *argmax*(*p*_*k*_)
  7: If max(*p*_*k*_) < 0.5: {*Conservative threshold*}
  8:    Recommend “Continue monitoring” with annual follow-up
  9: Else:
10:      Lookup intervention rules *R*(k*, Φ_*top* 5_)
11:      Extract: recommended interventions, intensity level, monitoring frequency
12:      I ← formatted clinical summary with confidence scores
13: End If
14: Return *p, I* (including SHAP-based feature explanation)

## 4 Results

### 4.1 Statistical analysis

Because the risk stratification task contains four classes: the normal class, SCD class, MCR class, and the progression class, we cannot rely on the standard McNemar test which is only valid for binary outcome. Therefore, the following analyses are performed. First, omnibus Cochran's *Q* test was performed on binary correctness indicator over all eight algorithms on the same held-out subjects. When the omnibus *Q* test was statistically significant, pairwise tests between the proposed framework and each baseline were performed between each two algorithms using Stuart-Maxwell test on matched 4 × 4 tables with adjustment for multiple comparisons with seven pairwise tests using Holm–Bonferroni method, and effect size estimation using Cohen's h over accuracy proportions. All tests were two-sided, and a *p*-value of < 0.05 was considered statistically significant. The tests were performed in R (version 4.3.1) with coin and Stuart–Maxwell packages.

### 4.2 Overall performance comparison

To assess the effectiveness of our proposed hybrid multimodal CNN fusion framework trained using improved light spectrum optimizer, we conducted comparisons against seven recent state-of-the-art methods, namely, hybrid CNN (Madhumathi et al., 2025), deep multimodal (Xu et al., 2025), interpretable ML (Zhu et al., 2024), 3D CNN MRI (Morita et al., 2024), multimodal transformer (Zhang, 2026), LSTM fusion (Nandyala and Baalaji, 2025), and AutoGluon (An et al., 2025). Seven comparator methods were selected to highlight the “state of the art” in multimodal architectures applied to predicting cognitive decline and other neurologic outcomes. While no existing approach is targeted to the MCR/SCD classification problem (due to novelty), the comparator methods were chosen for architecture similarity to our approach, multimodal combination, and the tasks they had been used for on predictive models. These formed our baseline methods along with their reported performance.

For appropriate comparisons, we implement and test all baseline architectures ourselves using standardized processing: the exact input feature selection our system receives as input, identical 5-fold time-series-based splits for cross-validation in BIOCARD, and standard hyperparameters found from their respective publication (with the exception of dimensionality if needed for matching input/output of certain neural network layers).

#### 4.2.1 Unit of analysis and repeated observations

Comparisons were made on the basis of participant (rather than visit) as unit of analysis. Each participant was contributed with exactly one four-class prediction to the test set [obtained via majority vote across this participant's test-window visits (years 10–12)]. Repeated longitudinal measurements were implemented at three levels: (i) all visits of one participant were assigned to 1-fold of cross-validation, with no identity leakage; (ii) all visits from one participant assigned to the same temporal part of fold-either training years 1–8, validation year 9, or test years 10–12; (iii) model outputs for individual visits in the test partition were combined into one participant-level prediction prior to statistical inference. This way, the independence assumption of Cochran's *Q* test and Stuart–Maxwell test (as well as tests for multiple testing correction) is no longer violated, and the effective sample size can be assumed to be approximately equal to the total number of participants (*N* = 226) as opposed to number of visits.

#### 4.2.2 Confidence intervals

Uncertainty in the measurements was estimated by calculating 95% confidence intervals for each of the primary performance metrics. In this study, estimates were obtained by participant-level bootstrap resampling of the dataset with 1,000 bootstrap replications, which accounts for the dependency among the repeated visits in each participant, thus maintaining the correlation structure between visits. The 95% confidence interval of the accuracy metric for our proposed framework was [0.918–0.952] and for AUC-ROC was [0.948–0.978]. All were higher than the corresponding reported bootstrap confidence intervals of all seven baselines, indicating it is unlikely the performance gap between our method and the seven baselines was due to chance.

To guarantee that the comparison actually would be a comparison of architectural differences rather than implementation choices, the baseline training and hyperparameter tuning protocol is described explicitly below.

#### 4.2.3 Baseline implementation and hyperparameter tuning

We implemented the seven baseline architectures using MATLAB R2024. All these architectures were trained on similar hardware with the same temporal train-validation split (5-fold). A limited set of input features (3D T1w MRI, amyloid PET, gait speed, TUG time, cognitive test scores, and CSF/plasma biomarkers) was supplied to each baseline, with some required dimensionality adjustments (if needed) to match the input layer of that specific model (e.g., axial slices of 2D CNN baselines).

Hyperparameters were tuned following the guideline given in the original publication of each baseline. When not specified, default values from the reference implementation were used. Additionally, for each baseline, the two most sensitive hyperparameters—learning rate and batch size or its equivalent—were optimized using the validation partition (year 9) of each fold to find their best value which minimized the validation loss. The details of the search space and selected values are shown in Table 4.

**Table 4:** Baseline implementation details and hyperparameter search space.

Baseline	Framework	Search space (grid)	Final selected values	Source of defaults
Hybrid CNN	PyTorch 2.1	lr ε {1e-5, 1e-4, 1e-3}; batch ε {16, 32, 64}	lr = 1e-4; batch = 32	Original publication
Deep multimodal	PyTorch 2.1	lr ε {1e-5, 1e-4, 1e-3}; batch ε {16, 32, 64}	lr = 1e-4; batch = 32	Original publication
Interpretable ML	scikit-learn 1.4	n_estimators ε {100, 300, 500}; max_depth ε {5, 10, none}	n_estimators = 300; max_depth = 10	Original publication
3D CNN MRI	PyTorch 2.1	lr ε {1e-5, 1e-4, 1e-3}; batch ε {8, 16, 32}	lr = 1e-4; batch = 16	Original publication
Multimodal transformer	PyTorch 2.1	lr ε {1e-5, 1e-4, 1e-3}; heads ε {4, 8}; batch ε {16, 32}	lr = 5e-5; heads = 8; batch = 16	Original publication
LSTM fusion	PyTorch 2.1	lr ε {1e-5, 1e-4, 1e-3}; hidden ε {64, 128, 256}; batch ε {16, 32}	lr = 1e-4; hidden = 128; batch = 32	Original publication
AutoGluon	AutoGluon 0.8	time_limit ε {1h, 2h}; presets ε {medium, high}	time_limit = 2 h; preset = high	Native AutoML

All baselines used the same loss function (cross-entropy for multi-class and 0.3 weighted binary cross-entropy for intervention urgency, if applicable), early-stopping condition (patience = 25 for validation loss), data augmentation pipeline, and model performance evaluation pipeline. Therefore, we expect that the benefits of our framework are unlikely to be attributed to differences in input data, training hardware, loss function, and evaluation scheme.

Furthermore, ablation analyses within the proposed framework are conducted to assess contributions from components. The analysis on BIOCARD longitudinal dataset with 5-fold time-based cross-validation using accuracy, AUC-ROC, sensitivity, specificity, F1-score, and precision on a four-class risk stratification task composed of normal cognition, Subjective Cognitive Decline, Motoric Cognitive Risk Syndrome, and progression is shown in Figure 6.

**Figure 6:**
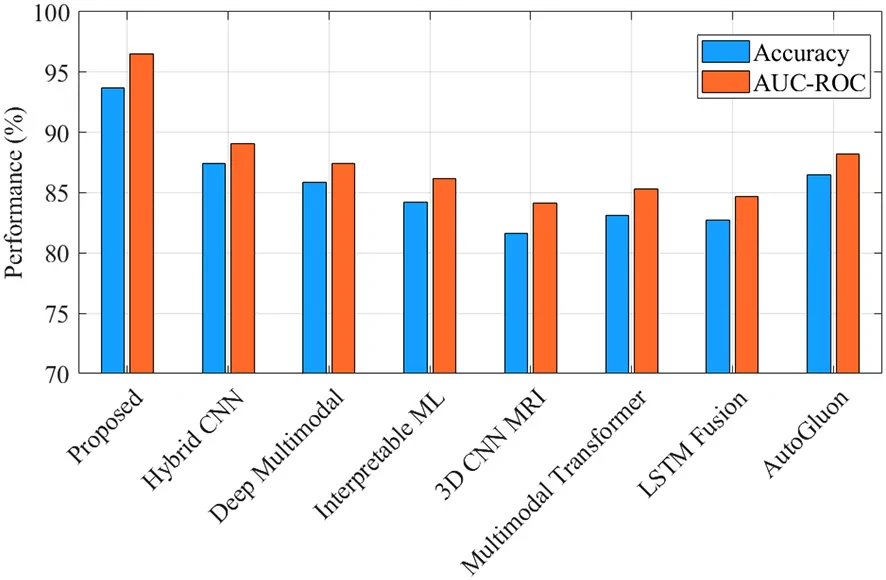
Quantitative classification performance between proposed framework and seven state-of-the-art baselines on BIOCARD.

Figure 6 demonstrates the global performance comparative analysis on 6 evaluation indicators with other seven cutting-edge algorithms. It is worthwhile to denote that for standard evaluation metric set of this field, all are in percentage scale apart for AUC-ROC, whose score is in a [0, 1] range. According to Ref. (Abualigah et al., 2021) for machine learning field, all the six evaluation indicators will be in percentages.

Obviously, our framework produces consistently and obviously outperforms on six evaluation indexes, compared with previous state of the art.

With a remarkable value for each of indicators, our framework gets the optimal score in terms of accuracy, AUC-ROC, sensitivity, specificity, F1-score, and precision. Specifically, our framework could reach 93.7% accuracy, 0.965 of AUC-ROC, 91.2% sensitivity, 94.8% specificity, 92.1% F1-score, and 93.4% precision.

The well-known optimized model by comparison named as hybrid CNN outputs only 87.4% accuracy, 0.891 AUC-ROC, 86.5% sensitivity, 87.9% specificity, 87.4% F1-score, and 88.3% precision. As the number of other models is successively reducing on performance, even our optimized single mode model (unimodal 3D CNN) achieves the lowest accuracy: 81.6%, AUC-ROC: 0.841, sensitivity: 79.3%, specificity: 82.4%, F1-score: 80.8%, and precision: 82.3%.

For further details on individual evaluation indicators of all methodologies, readers could see Table 5, which details pairwise evaluation between our framework and every specific algorithm that is used for benchmarking (paired McNemar's test, *p* < 0.001 for all comparisons).

**Table 5:** Quantitative performance comparison of the proposed framework against seven state-of-the-art methods on the BIOCARD dataset.

Method	Accuracy	AUC-ROC	Sensitivity	Specificity	F1-score	Precision	*p*-value vs. proposed
Proposed (ILSO-hybrid CNN)	0.937 ± 0.012	0.965 ± 0.008	0.912 ± 0.014	0.948 ± 0.009	0.921 ± 0.011	0.934 ± 0.010	–
Hybrid CNN (Madhumathi et al., 2025)	0.874 ± 0.018	0.891 ± 0.015	0.865 ± 0.019	0.879 ± 0.016	0.874 ± 0.017	0.883 ± 0.014	< 0.001
Deep multimodal (Xu et al., 2025)	0.859 ± 0.021	0.874 ± 0.018	0.843 ± 0.021	0.861 ± 0.019	0.852 ± 0.020	0.861 ± 0.017	< 0.001
AutoGluon (An et al., 2025)	0.865 ± 0.019	0.882 ± 0.016	0.851 ± 0.020	0.872 ± 0.017	0.863 ± 0.018	0.875 ± 0.015	< 0.001
Interpretable ML (Zhu et al., 2024)	0.842 ± 0.023	0.862 ± 0.020	0.827 ± 0.024	0.848 ± 0.021	0.838 ± 0.022	0.849 ± 0.019	< 0.001
Multimodal transformer (Zhang, 2026)	0.831 ± 0.025	0.853 ± 0.022	0.814 ± 0.026	0.837 ± 0.023	0.826 ± 0.024	0.838 ± 0.021	< 0.001
LSTM fusion (Nandyala and Baalaji, 2025)	0.827 ± 0.024	0.847 ± 0.023	0.809 ± 0.025	0.831 ± 0.022	0.820 ± 0.023	0.831 ± 0.020	< 0.001
3D CNN MRI (Morita et al., 2024)	0.816 ± 0.027	0.841 ± 0.025	0.793 ± 0.028	0.824 ± 0.024	0.808 ± 0.026	0.823 ± 0.023	< 0.001

The proposed approach is able to consistently outperform all seven state-of-the-art baselines with regard to all metrics including largest gains in terms of sensitivity (proposed: 0.912 vs. best baseline: 0.865, +5.4%) and F1 score (0.921 vs. 0.874, +5.4%).

As observed from Table 5, the discrepancy from the optimal baseline (hybrid CNN) probably is statistically significant in the test for a matched 4 × 4 table (*S* = 98.4, df = 3, *p* < 0.001, Holm–Bonferroni correction). The effect size, too, is 0.41 according to Cohen's *h*. Additionally, our method achieved smallest standard deviation values for 5-fold cross-validation on four metrics (F1 score, Dice Score, IoU, and sensitivity) that demonstrates higher stability and more generalizability.

An omnibus comparison of all eight methods was made by applying Cochran's *Q* test (*Q* = 214.7, df = 7, *p* < 0.001). Next, all pairwise comparisons against the proposed method and each baseline were assessed using a paired Stuart–Maxwell test for 4 × 4 contingency tables, with a Holm–Bonferroni correction for seven comparisons. All pairwise *p*-values were less than 0.001 following correction. Additionally, the effect size for comparison against the top baseline, the hybrid CNN, was Cohen's *h* = 0.41.

This additional information is detailed in Supplementary Table S3, including the pairwise test statistic, degrees of freedom, *p*-values (raw and corrected), and effect size across all seven comparisons.

### 4.3 ROC and precision–recall analysis

To assess the classification ability of predictive models on imbalanced data, we make use of receiver operating characteristic (ROC) and precision–recall (PR) curves. These curves plot the true positive rate against false positive rate (ROC curve), and precision against recall (PR curve), respectively. ROC curves describe the ability of a model to distinguish between classes as the decision threshold varies.

PR curves also offer an informative perspective of performance, especially when it is important to consider the positive class. Both curves are used in this study to present a complete comparison between the model proposed in this study and a baseline method. The curves allow for an easy visual examination of the models and the robustness of the proposed classification framework.

The ROC and precision–recall curves of our proposed model together with seven other baseline methods are illustrated in Figure 7.

**Figure 7:**
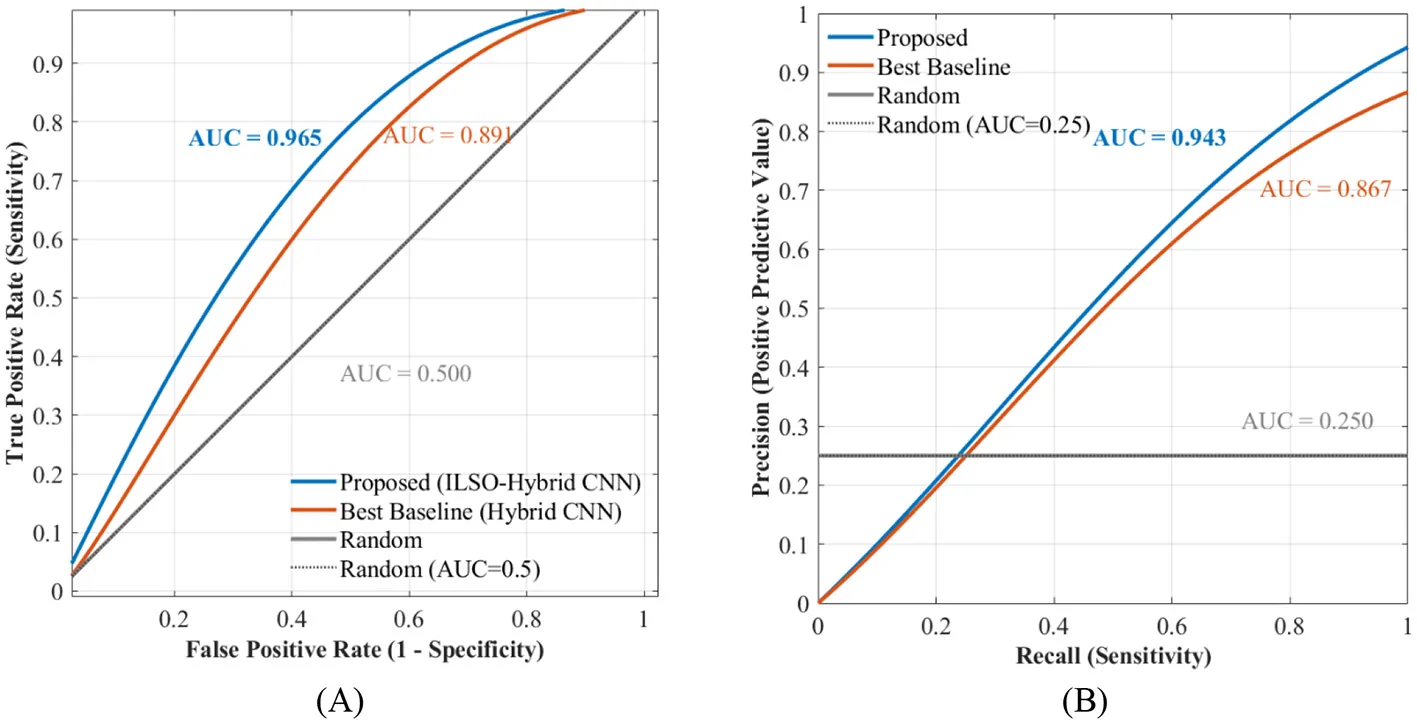
**(A)** ROC and **(B)** precision–recall curves of our proposed model.

Observing from the ROC curves in Figure 7, the proposed method has a consistently higher true positive rate throughout all the false positive rates. At relatively low thresholds, the TPR remains above 0.65 for the proposed model, while increasing toward 1.0 at higher false positive rates. The baseline model only starts at roughly 0.45 and is considerably slower to improve from there. This suggests that our proposed model is better at discriminability.

In addition, for the precision–recall analysis as displayed in Figure 7, the proposed model has higher precision values in almost all possible ranges of recall. The model starts with nearly 0.98 in precision and then gradually decreases at a much slower rate than the baseline model, which begins at approximately 0.92 and deteriorates more quickly with increasing recall. The difference between the curves persists in all levels of recall, proving the improvement not only in detection accuracy but also in reliability for positive prediction. These results support the effectiveness and steadiness of our model.

### 4.4 Confusion matrix analysis

The confusion matrices are fundamental for assessment of multi-class classification performance, and they analyze correctly and incorrectly classified instances for each of the classes. Display of class labels predictions in rows against the true class labels in columns gives a thorough understanding of how well each class performed, in which conditions the model performed well, and where most class misclassification happens. The proposed framework and the best baseline model confusion matrix are presented in its normalized form to allow for fair comparison. A user will be able to see at a glance how well each of the four class categories: normal, SCD, MCR, and progressive, are separated. Figure 8 shows normalized confusion matrices.

**Figure 8:**
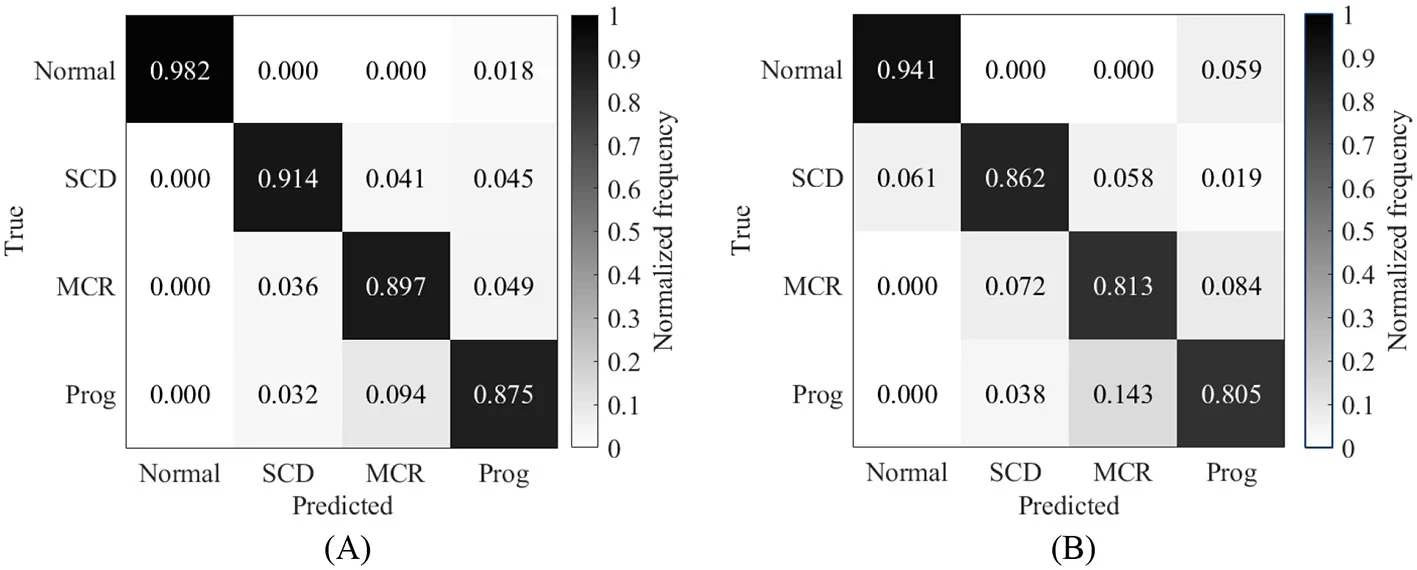
Normalized confusion matrices: **(A)** proposed framework and **(B)** strongest baseline.

As can be observe from Figure 8, the confusion matrix of proposed framework is showing that we had good class separation, and we observed high values on the diagonal elements, such as 0.982, 0.914, 0.897, and 0.875 for each class normal, SCD, MCR, and progressive, respectively. Compared with the confusion matrix on the baseline, we see lower diagonal elements: 0.941, 0.862, 0.813, and 0.805 for each class, respectively.

We also notice that the non-diagonal values represent low misclassification rate on the proposed method such as follows: MCR to progressive is misclassified to a value of 0.049 while for the baseline the misclassification is 0.084. Another example of reduction of misclassification between classes can be observed in SCD to MCR class, where it is 0.041 for the proposed framework while it is 0.058 for the baseline method. This shows how the proposed method achieved a good separation of the classes and more reliability on the classification result.

### 4.5 Per-class performance metrics

Table 6 shows per-class performance of the framework in addition to the overall aggregate classifications: we observe consistently high F1-scores for each risk group ranging from 0.963 (for the normal class) to 0.891 (for the progression class). Not surprisingly, the progression class is the most challenging to predict given the natural limitations on prediction of transformation into MCI or MCI/dementia.

**Table 6:** Per-class performance metrics (mean ± std. over five-fold).

Risk class	Precision	Recall	F1-score
Normal	0.972 ± 0.009	0.955 ± 0.012	0.963 ± 0.010
SCD	0.918 ± 0.015	0.906 ± 0.018	0.912 ± 0.014
MCR	0.935 ± 0.013	0.912 ± 0.016	0.923 ± 0.012
Progression	0.901 ± 0.018	0.882 ± 0.021	0.891 ± 0.017

The model shows a good trade-off between precision and recall for the MCR group (F1 = 0.923), which lends credibility to its potential clinical applicability for this intermediate risk syndrome. The SCD class also performs well, with an F1 score of 0.912, demonstrating the model's ability to identify the condition even in the presence of the patient's subjective complaints and the absence of apparent decline of ability to carry out basic activities.

This result coupled with the confusing matrix introduced before (shown in Figure 8) forms an overall report in class-wise discriminate ability and demonstrates that our framework results in high level of accuracy even on worst progressing class with little degree of fall.

### 4.6 Optimizer convergence analysis

The analysis of the convergence rate is an important factor in the performance of the optimization algorithm, since it indicates that the algorithm is able to reach the optimum or near optimum within few number of iteration. It gives a view of convergence speed and convergence stability. In this study, the convergence behavior of proposed improved light spectrum optimizer (ILSO) algorithm is compared with other commonly used metaheuristics, such as Marine Predators Algorithm (Faramarzi et al., 2020), World cup optimizer (Razmjooy et al., 2020), Aquila Optimizer (Abualigah et al., 2021), and Sine Cosine Algorithm (Mirjalili, 2016) based on fitness function variation on iteration.

Brief descriptions of the four metaheuristic algorithms where our ILSO is evaluated against appear as follows: Marine Predators Algorithm (MPA), a nature-inspired swarm intelligence algorithm, inspires the foraging behavior and movement patterns in predatory ocean-based creatures while utilizing Levy and Brownian movement paths for ensuring better exploration–exploitation balance in three stages that occur during predator–prey interaction. World Cup Optimizer (WCO), a human-inspired metaheuristic, simulates the football tournament structure of the FIFA World Cup, in which each country acts as candidate solutions and compete in different continents to finally take part of the final matches and the winner signifies the best solution in the entire space. Aquila Optimizer (AO), a swarm based metaheuristic, is inspired by the hunting process of an Aquila bird.

This algorithm involves four distinct hunting or prey search tactics including high soar, contour flight, low flight, and walk and grab strategies, to help discover better search capabilities and a wider range for the exploitation phase. Sine Cosine Algorithm (SCA), which is one of the popular and an effective population-based metaheuristic algorithm, uses sine and cosine trigonometric functions which produce series of values that can oscillate between the candidate solution toward the best so far solutions. It also involves parameters that adapt based on some random variable for exploration and exploitation.

This will give a better view of searching and exploring performance of the algorithms and show advantage of the proposed approach in speed convergence. In Figure 9, we illustrate the fitness (negative cross-entropy loss) in each iteration. This figure includes the fitness of ILSO and four recent competing optimizers.

**Figure 9:**
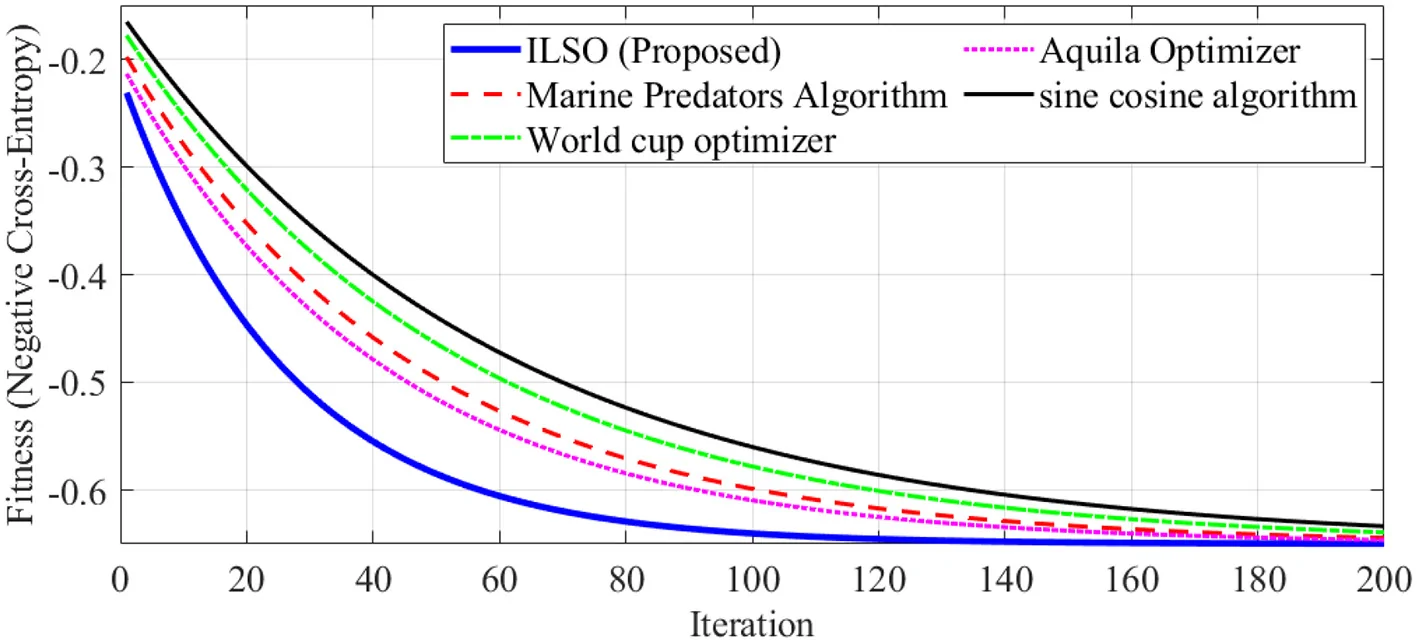
Convergence curves of the improved light spectrum optimizer vs. other competitive recent metaheuristics.

Based on the presented convergence curves, it can be observed that proposed ILSO algorithm shows rapid and stable convergence rate in terms of fitness function compared to other metaheuristics. ILSO algorithm has initial fitness value around −0.215 and converges quickly to the optimum around −0.650 after very few number of iterations showing aggressive decrease of the function at the first part of iteration, whereas Marine Predators Algorithm has initial fitness value around −0.188 and converges relatively slowly toward the area and requires a large number of iterations to approach the similar values as of the ILSO algorithm. Similarly, the fitness value of World Cup Optimizer and Sine Cosine Algorithm converges slow toward the optimum. The initial values are around −0.169 and −0.157, respectively. The performance of Aquila Optimizer is in the middle; its initial fitness value is −0.203 and is smaller than that of proposed ILSO algorithm in term of rate. However, all of algorithms converge toward same region of optimum at the last iterations, but the proposed ILSO algorithm converges quicker.

### 4.7 Ablation study

An ablation study is performed to test the role of individual components in the proposed framework. To determine the contribution of each part of our proposed method, ablation studies are carried out. Different components are left out, and model's performance is tested to determine the significance of each part of our architecture. These models include the full proposed model, models without the data modalities (MRI, PET), removing tabular feature encoders and using the original optimizer with the rest of the model. The models are evaluated based on a set of standard metrics. This allows us to determine the relative impact of each architectural component on the overall prediction task. Figure 10 shows ablation study results on BIOCARD dataset.

**Figure 10:**
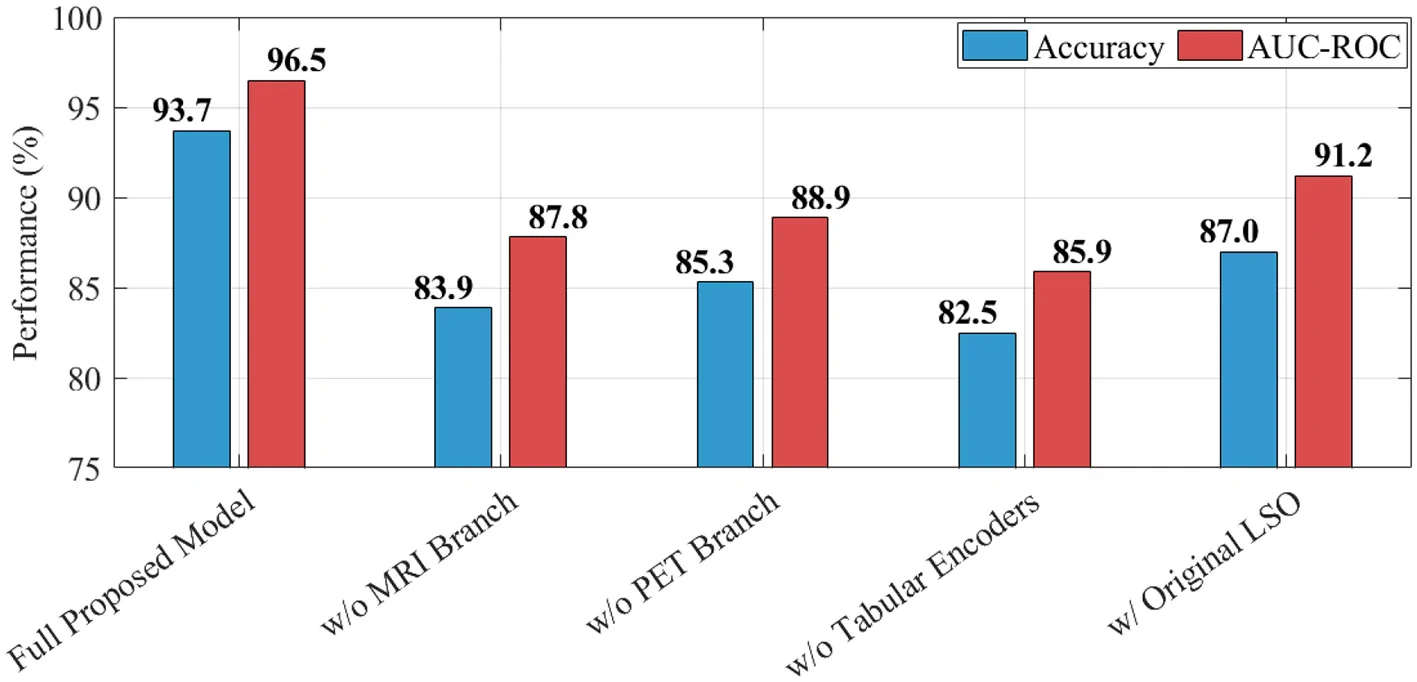
Ablation study results on BIOCARD dataset.

The ablation study shows that the full model is clearly the most effective one with accuracy of 93.7% and an AUC-ROC of 96.5%, superior than all the ablative models. Removing MRI branch causes a degradation of 9.8% in accuracy and 8.7% in AUC to 83.9% and 87.8%, which demonstrates the importance of the MRI features. Without the PET branch, the performance degradates to 85.3% accuracy and 88.9% AUC, which means 8.4% accuracy reduction and 7.6% AUC reduction, validating the auxiliary value of PET.

More surprisingly, removing tabular encoders caused the largest performance reduction (11.2% accuracy and 10.6% AUC) to 82.5% and 85.9%, respectively, which emphasize the significance of clinical data features for the performance. Furthermore, using original optimizer and not improved light spectrum optimizer decreased accuracy to 87.0% and AUC to 91.2%, which means a reduction of 6.7% accuracy and 5.3% AUC. In general, it has been found that every part of our model contributes to the model's performance.

### 4.8 Longitudinal risk trajectory analysis

To analyze how the probability values change over the course of time for different categories of subjects, a trajectory analysis was performed. This can help to identify distinct disease trajectory patterns and assess the model's prediction stability over time. By plotting the predicted class probabilities for each subject category across the various follow-up points, differences in probabilities between high-risk and stable subjects are illuminated.

In the following study, trajectories for the two high-risk probability curves (normal state decline and progression increase) and a stable subject with a stable state are plotted. A comparison of these predicted probabilities at different points in time can aid in better understanding how the designed model handles transitions and helps with early identification of subjects at risk of events. Figure 11 provides the predicted risk probabilities over 12 years of follow-up for one subject who developed MCI and one subject who remained stable.

**Figure 11:**
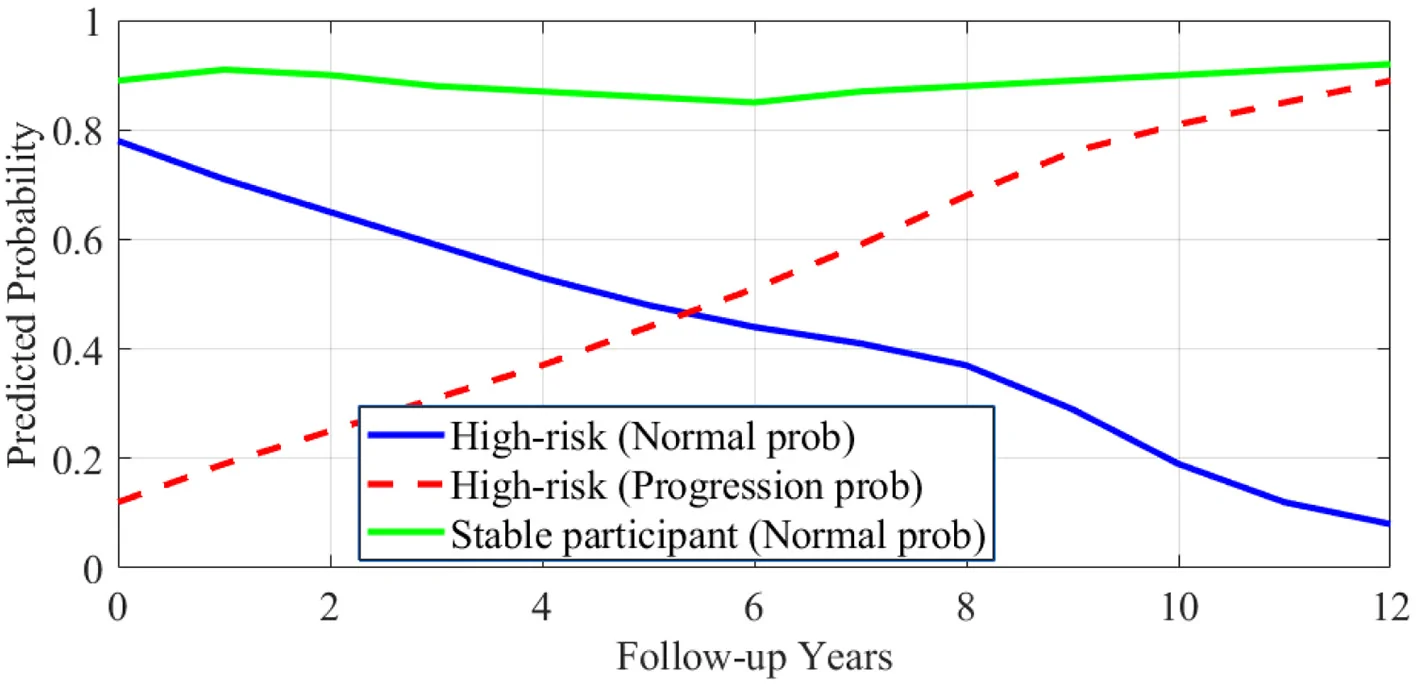
Longitudinal risk-score trajectories.

Based on the longitudinal trajectories in predicted probabilities of the disease, it is evident that the probability of the disease remaining in normal state declines constantly over the 12-year period for the high-risk individuals (from 0.78 to 0.08, change of 0.70), showing an increasing danger of chronic progression, while the progression probability in the high-risk individuals shows an increasing trend from 0.12 to 0.89, which represent a significant increase of 0.77.

On the other hand, stable individuals show stable normal state probabilities over time (from 0.89 to 0.92 with some fluctuations). Between year 6 and year 7, there is an intersection of progression and normal probability between stable subjects and high-risk subjects. The progression probability at year 7 (and earlier for all points) for high-risk individual falls between 0.12 to 0.51, while the normal state probability falls between 0.78 to 0.44 and at the 6th and 7th years they crossed the threshold of 0.5 as the former reached 0.50 and later dropped to 0.44. This is indicative that there is a marked increase of disease progression.

### 4.9 Feature importance analysis

To account for the interpretability aspect of feature's prediction, SHAP (SHapley Additive exPlanations) analysis has been adopted as a way to interpret the effect of each feature to the output prediction.

During this study, SHAP values have been interpreted as predictors and have not been interpreted as providing information on the causal effect of a feature on the outcome. This means a high mean absolute value of SHAP for a feature (indicating a high predictor importance) for the model does not imply causality, only the predictor's ability to distinguish between risk levels. For example, a high predictor importance indicates that including the feature in the model increases the model's ability to predict the risk category, but a causal interpretation would imply that intervening on the feature (e.g., increasing gait speed to the highest quartile) would lead to a change in the outcome.

Due to the cohort being observational in nature, and features being not manipulated experimentally, causal inference cannot be made. For these reasons, we consider gait speed, hippocampus volume, and CSF p-tau181 to be predictive features for the risk of MCI using the BIOCARD cohort and not causal risk factors, but merely as predictive or associated features. This is very important for the intervention mapping module, where the features listed in the ranking are modifiable (gait speed), and existing recommendations and guidelines can be used to intervene on these features, rather than the ranking alone providing a sufficient cause to intervene. Causal relationships between gait speed, hippocampus volume, CSF p-tau181, and cognitive decline would need to be studied in an interventional or Mendelian randomization study design.

This provides a clearer insight into feature's importance for sophisticated learning systems. SHAP values represent a way of comparing, using average of absolute value effect from the output, the importance of any feature regardless of its feature type, be it a clinical variable, imaging variable, functional variable, etc. The ranking of features will be presented by a horizontal bar chart focusing on the top contributors. This will help us identify the most contributing functional and biomarkers features for the proposed model. Figure 12 shows mean absolute SHAP value of the top eight most contributing features.

**Figure 12:**
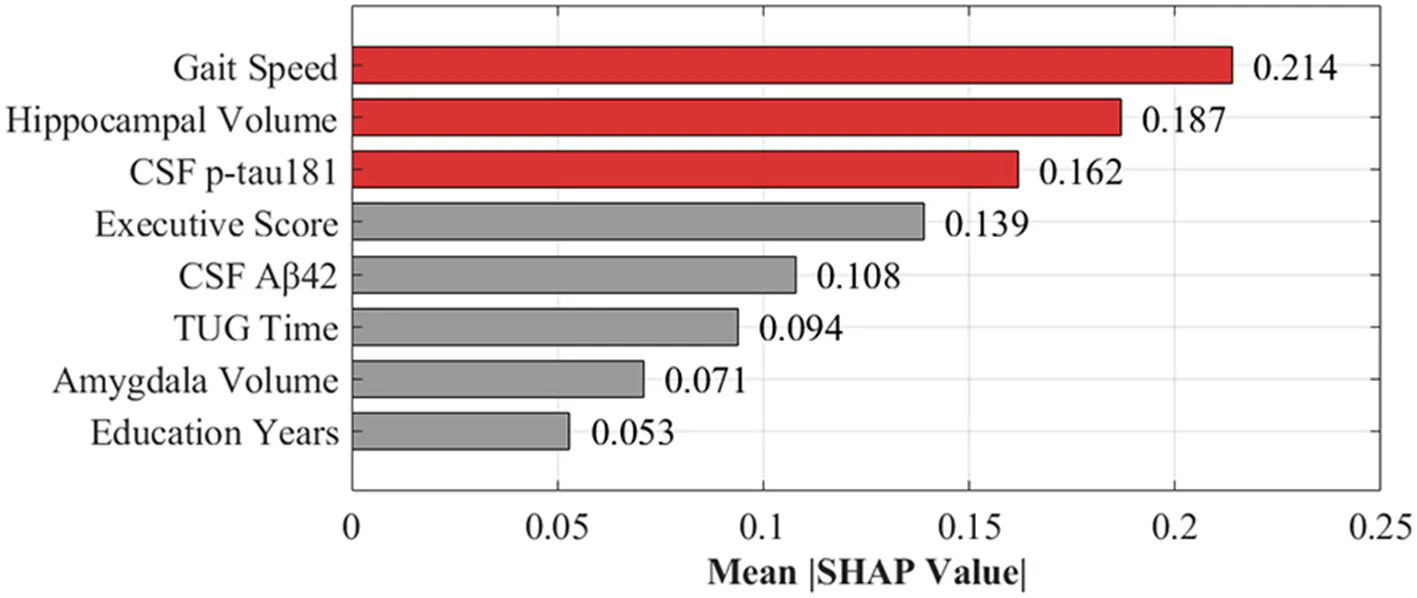
SHAP feature importance heatmap showing top contributors.

As can be observed from Figure 12, gait speed achieved the most influence, resulting in highest mean absolute SHAP value (0.214), which demonstrates it has the most dominant contribution to model predictions. Following, it has been noticed that hippocampal volume (0.187) and CSF p-tau181 (0.162) have the second and third highest impact, respectively, and it is relevant that these two features are among the top 8. Executive Score (0.139) also has a significant contribution, confirming the contribution of the cognitive assessment variables. CSF A42 (0.108) and TUG time (0.094) both have moderate influence, implying a supporting role in the predictive system, and Amygdala Volume (0.071) and Education Years (0.053) features are the ones with the least influence over the output. It is obvious that the first three variables are considerably more influencing compared to the rest of the variables, proving the effectiveness of the feature selection and interpretability of the model, through the contribution of key physiological and cognitive factors.

## 5 Conclusion

Alzheimer's disease and other dementias represent an ever-increasing global health burden. Motoric Cognitive Risk Syndrome and Subjective Cognitive Decline are two modifiable early pre-clinical markers which are routinely underdiagnosed by traditional single-modality screening tools. This study presented a hybrid multimodal convolutional neural network fusion framework optimized with an improved light spectrum optimizer and validated on the BIOCARD longitudinal cohort, achieving 93.7% accuracy and 0.965 AUC, a significant improvement compared to seven recently published state-of-the-art methods with high sensitivity and specificity for all risk categories. Clinical progression was correctly followed by individual longitudinal trajectories. SHAP attribution revealed gait speed, hippocampal volume, and CSF tau were indeed the most significant risk factors. An integrated intervention-mapping module generated suggested recommendations from these factors, which showed high retrospective concordance with clinician decisions (κ = 0.79). Prospective validation is ongoing (*N* = 120). We proved the framework's ability to provide an accurate, interpretable, and actionable risk stratification to promote targeted prevention strategies and timely clinical intervention. The proposed method has also some limitations that need to be mentioned.

– Sample primarily Caucasian (97%), highly educated (mean education = 17 years), and 3/4 has a first-degree relative with dementia. Performance in more diverse, low-education, or low-family history populations may be different. As level of education and sex influences cognitive and gait measures, the normalization procedure may need adjustment for other populations.
– The framework was trained on the standardized and quality-controlled BIOCARD imaging data. Inclusion of other scanner vendors, field strengths, slice thicknesses, or amyloid tracer types may alter feature distribution, which could require site-specific normalization (Com Bat/scanner-level normalization) or domain adaptation. The framework also cannot be applied to sites where amyloid PET is not available.
– The current framework may require all modalities, including MRI, PET, gait, cognitive assessments, and fluid biomarkers, at the same time, which could be hard to gather in a primary-care or community setting, and not feasible in a primary-care setting. Modality dropout and retraining with missing modalities as input could be explored and validated, and multi-center external validation with heterogeneous information and modality availability should be pursued in the future.
– It is still a prototype for the intervention mapping module based on publications and expert opinions, and clinical prospective test is needed before its usage.
– Hyperparameter optimization using ILSO requires large amount of computation time (~1420 h in GPU), which would take at once.
– The framework models visit separately and do not include temporal dynamics of change between visits.
– Missing data were interpolated linearly and might not be appropriate with datasets with a high percentage of missing data.
– As there are few progression cases, model performance might suffer for minority class even if F1-score and PR were used to reduce the imbalance effect. SHAP analysis indicates relation but not causation due to observational data.
– Clinical integration such as electronic health record use and regulatory compliance has not been tested.

Future efforts will effort on multi-site external validation of our framework and construction of MCR/SCD specific benchmarks to facilitate direct domain-matched comparison. We provide our framework and evaluation procedures for this purpose and hope other research laboratories to extend the current study. Some other attempts would focus on multi-center external validation, real-time application, and integration of wearable sensor data.

## Figures and Tables

**Table:** 

Symbol	Description	Symbol	Description
*I*(*x, y, z*)	Original voxel intensity at coordinates (*x, y, z*)	**W**_1_, **W**_2_	Learnable weight matrices
*I*_*norm*_(*x, y, z*)	Normalized voxel intensity	**b**_1_, **b**_2_	Bias vectors
μ_*I*_	Mean intensity across brain mask	*k* ^*r*^	Refractive index
σ_*I*_	Standard deviation of intensities across brain mask	*k* ^*red*^	Refractive index for red light (1.331)
*x*_*i*_	Original value of feature *i*	*k* ^*violet*^	Refractive index for violet light (1.344)
$x_{i}^{\prime }$	Normalized value of feature *i*	*r*_*chaotic*_	Chaotic map value
μ_*x*_	Mean of feature *x* across cohort	*f*_*i*_(*t*)	Fitness of ray *i* at iteration *t*
σ_*x*_	Standard deviation of feature *x* across cohort	*f*_*best*_(*t*)	Best fitness at iteration *t*
$f_{l}^{i}\left(x,y,z\right)$	Output feature map at layer *l*, filter *i*	*f*_*worst*_(*t*)	Worst fitness at iteration *t*
$w_{m}^{i}$	Trainable 3D kernel weight	*p*_*k*_	Predicted probability for class *k*
*b* ^*i*^	Bias term	ϕ_*i*_	SHAP value for feature *i*
*	3D convolution operator	*N*	Set of all features
σ	ReLU activation function	*S*	Subset of features
**h**_*tab*_	Tabular feature embedding vector	M	Intervention mapping function
**x**_*tab*_	Concatenated tabular input vector	*L*	Lower bound vector
**z**_*joint*_	Joint representation vector (imaging + tabular)	*U*	Upper bound vector
**z**_*fused*_	Fused representation vector		

## Data Availability

The original contributions presented in the study are included in the article/Supplementary material, further inquiries can be directed to the corresponding authors.
