# Supplementary material for: An AI-driven model for risk stratification and prototype intervention mapping in motoric cognitive risk syndrome and subjective cognitive decline
Source: Front Aging Neurosci. 2026 Oct 23;18:1878389. doi: 10.3389/fnagi.2026.1878389 (PMC13739817; doi:10.3389/fnagi.2026.1878389)
Supplement: Supplementary file 1 [file Supplementary_file_1.docx]

**Supplementary materials**

Table S1. Comprehensive architectural and training specifications

| Category | Specification |
| --- | --- |
| MRI 3D CNN | 4 conv blocks: filters [32,64,128,256], kernel 3³, stride 1, padding same; batch norm + ReLU + max‑pool (2³, stride 2) after each; global avg‑pool after block 4 → 256‑D, FC to 512‑D + dropout 0.4 |
| PET 3D CNN | Identical architecture to MRI branch (shared parameters) |
| Motor Encoder | FC layers [256,128,64] + batch norm + ReLU + dropout 0.3; output 64‑D |
| Cognitive Encoder | Same as motor encoder |
| Biomarker Encoder | Same as motor encoder |
| Joint Fusion | Concatenate (MRI 512 + PET 512 + tabular 192) → 704‑D; FC 1024 (ReLU + BN + dropout 0.35) + residual connection; FC 512 (ReLU + BN + dropout 0.3) → fused 512‑D |
| Classification Head | FC 256 (ReLU) → softmax 4‑class; separate sigmoid branch for intervention urgency |
| Weights Initialization | He uniform (ReLU); Glorot uniform (sigmoid/softmax) |
| Optimizer | Adam (lr=1×10⁻⁴, β₁=0.9, β₂=0.999, ε=1×10⁻⁸, weight decay=1×10⁻⁵) |
| LR Schedule | Cosine annealing from 1×10⁻⁴ to 1×10⁻⁶ over 150 epochs with 10‑epoch warm‑up |
| Loss Function | L_total = cross‑entropy + 0.3 × binary‑cross‑entropy (urgency) |
| Batch Size | 32 (with 2‑step gradient accumulation → effective 64) |
| Epochs and Early Stop | Max 200; patience 25 (validation loss) |
| Data Augmentation | Imaging: random rotations (±10°), translations (±5 mm), elastic deform (α=0.1,σ=2) with p=0.5; tabular: Gaussian noise (σ=0.01) with p=0.3 |
| Cross‑Validation | 5‑fold time‑based (train years 1-8, val year 9, test years 10-12) |
| ILSO Hyper‑search | Population 30, max iterations 100; adaptive refractive index (Eq. 8), chaotic map (Eq. 9), opposition‑based learning every 10 iters; fitness = validation loss |
| Search Space | Filters [16 512], kernels [3³,5³,7³], dropout [0.1 0.6], lr [1×10⁻⁵ 5×10⁻^4^], L2 [1×10⁻^6^ 1×10⁻^3^], fusion dims [256 2048], batch [16 128] |
| Final Optimized Values | Filters [64, 128, 256, 512]; kernel 3³; dropout 0.35/0.40/0.30 (fusion/imaging/tabular); lr=1.2×10⁻⁴; L2=8.7×10⁻⁶; fusion dims 1024, 512; batch=32 |
| Hardware/Software | NVIDIA A100 (80GB), MATLAB R2024b |

where, all numbers in this section are average ± std across five folds, and a final ensemble results in major voting.

Table S2. Sensitivity analysis of imputation strategies

| Strategy | Accuracy | AUC-ROC | Sensitivity | Specificity | F1 | Precision |
| --- | --- | --- | --- | --- | --- | --- |
| Linear interpolation (primary) | 0.937 ± 0.012 | 0.965 ± 0.008 | 0.912 ± 0.014 | 0.948 ± 0.009 | 0.921 ± 0.011 | 0.934 ± 0.010 |
| LOCF  (forward only) | 0.934 ± 0.013 | 0.962 ± 0.009 | 0.909 ± 0.015 | 0.945 ± 0.010 | 0.918 ± 0.012 | 0.931 ± 0.011 |
| Complete-case analysis | 0.939 ± 0.014 | 0.966 ± 0.009 | 0.914 ± 0.016 | 0.950 ± 0.011 | 0.923 ± 0.012 | 0.936 ± 0.011 |
| *p-value*  *(Cochran's Q)* | 0.34 | 0.41 | 0.52 | 0.38 | 0.29 | 0.44 |

Table S3. Operational definitions of the four outcome categories.

| Category | Subjective complaint | Objective cognition | Gait speed | Functional status | CDR | Additional notes |
| --- | --- | --- | --- | --- | --- | --- |
| Normal | Absent | Within 1.5 SD of norms | Normal | Intact | 0 | Includes BIOCARD “Impaired Not MCI” |
| SCD | Present (persistent, self-experienced) | Within 1.5 SD of norms | Normal | Intact | 0 | SCD-I criteria; not explained by other conditions |
| MCR | Present | Within 1.5 SD of norms | ≥ 1 SD below age- and sex-adjusted mean | Intact | 0 | No dementia; no mobility disability |
| Progression (MCI) | Present | ≥ 1.5 SD below norms in ≥ 1 domain | Variable | Largely intact | 0.5 | NIA-AA criteria; first visit meeting criteria |
| Progression (Dementia) | Present | ≥ 1.5 SD below norms | Variable | Impaired (ADL) | ≥ 0.5 | Cognitive decline interferes with independence |

*where, CDR = Clinical Dementia Rating, MCI = Mild Cognitive Impairment, ADL = activities of daily living, SD = standard deviation.*

Table S4. The results of 2-sample Stuart-Maxwell Test of the proposed framework versus the baselines

| Comparison | Stuart-Maxwell S | df | Raw p | Holm-Bonferroni corrected p | Cohen's h | Interpretation |
| --- | --- | --- | --- | --- | --- | --- |
| Proposed vs. Hybrid CNN [25] | 98.4 | 3 | < 0.001 | < 0.001 | 0.41 | Medium-to-large effect |
| Proposed vs. Deep Multimodal [26] | 112.6 | 3 | < 0.001 | < 0.001 | 0.48 | Medium-to-large effect |
| Proposed vs. AutoGluon [31] | 105.2 | 3 | < 0.001 | < 0.001 | 0.45 | Medium-to-large effect |
| Proposed vs. Interpretable ML [27] | 128.9 | 3 | < 0.001 | < 0.001 | 0.54 | Large effect |
| Proposed vs. Multimodal Transformer [29] | 141.3 | 3 | < 0.001 | < 0.001 | 0.59 | Large effect |
| Proposed vs. LSTM Fusion [30] | 147.8 | 3 | < 0.001 | < 0.001 | 0.62 | Large effect |
| Proposed vs. 3D CNN MRI [28] | 162.4 | 3 | < 0.001 | < 0.001 | 0.68 | Large effect |

We use a matched 4 × 4 contingency table for all tested matched held-out subjects and measure predicted classes (Normal, SCD, MCR, Progression). The omnibus Cochran's Q test of all eight methods results in statistical significance (Q=214.7, d.f=7, p<0.001). Raw p-values are directly obtained from results of the Stuart-Maxwell test (d.f. = 3), with results being Holm-Bonferroni corrected for all seven pairwise comparisons. Effect sizes are obtained as Cohen's h on the proportions of the accuracy rates. All statistical tests are considered two-sided with an alpha of 0.05.

Algorithm 1: Hybrid multimodal CNN fusion framework training with ILSO

Input: BIOCARD longitudinal dataset $D = \left\{ \left( X_{MRI}, X_{PET}, X_{tab}, y, time \right) \right\}$

Preprocessed imaging, tabular, and label data

ILSO population size $N$, max iterations $T_{max}$ epochs $E$

Output: Optimized trained model with best hyperparameters $\Theta^{*}$

1: Split D into 5 temporally ordered folds (train years 1-8, val year 9, test years 10-12)

2: For fold = 1 to 5:

3: $D_{train}, D_{val}, D_{test}\leftarrow fold split$

4: Initialize ILSO population P with N candidate hyperparameter vectors

5: For $t = 1$ to $T\_max$:

6: For each candidate $\theta\in P$:

7: Initialize model architecture $M(\theta)$ with hyperparameters $\theta$

8: Train $M$ for 50 warm-up epochs on $D_{train}$ with Adam optimizer

9: Evaluate validation loss $L_{val}$ on $D_{val}$

10: $fitness\left( \theta\right)\leftarrow L_{val} \left\{ fitness = negative cross-entropy \right\}$

11: End For

12: Update population P via ILSO adaptive refractive index (Eq. 8)

13: Apply chaotic perturbation (Eq. 9) and opposition-based learning (Eq. 11)

14: End For

15: $\Theta* \leftarrow hyperparameter vector with best validation fitness$

16: Initialize final model $M^{*}$ with $\Theta^{*}$, train from scratch for $E=200$ epochs on $D_{train}\cup D_{val}$

17: Evaluate on $D_{test}$, store metrics

18: End For

19: Report mean ± std metrics across 5 folds

20: Return ensemble of 5 trained models (majority voting)

Algorithm 2: Improved light spectrum optimizer (ILSO)

Input: Search space bounds $[L, U]$ for each hyperparameter

Population size $N$, max iterations $T_{max}$ Chaotic map initial value $r_{chaotic\left( 0 \right)}= 0.7$

Output: Best hyperparameter vector $\Theta^{*}$

1: Initialize population $P = \left\{ x_{i} \right\}for i = 1,\ldots,N$ using Eq. (6)

2: Evaluate fitness $f\left( x_{i} \right)$ for each candidate (Algorithm 1, lines 7-10)

3: For t = 1 to $T_{max}$4: Identify best $x_{best}$ and worst $x_{worst}$ from current population

5: For each candidate $x_{i}$:

6: Compute fitness-adaptive refractive index $k_{r}\left( t \right)$ using Eq. (8)

7: Generate three normal vectors (random, current, global best)

8: Compute direction cosines using Snell's Law

9: If $rand < 0.5$: $\left\{ Global search phase \right\}$

10: Update using refraction rule with chaotic perturbation Eq. (10)

11: Else: $\left\{ Local search phase \right\}$

12: Perturb $x_{i}$ around $x_{best}$ via cosine-law reflections

13: End If

14: End For

15: If t mod 10 == 0: $\left\{ Opposition-based refinement \right\}$

16: For each $x_{i}$:

17: $x_{i}^{opp}= L + U - x_{i}$ (Eq. 11)

18: Keep fitter of $x_{i}$ and $x_{i}^{opp}$

19: End If

20: Update chaotic map: $r_{chaotic\left( t+1 \right)}= 4 \times r_{chaotic\left( t \right)}\times\left( 1 - r_{chaotic\left( t \right)} \right)$

21: End For

22: $\Theta* \leftarrow x_{i}$ with minimum fitness (lowest validation loss)

23: Return $\Theta^{*}$

Algorithm 3: Risk stratification and personalized intervention mapping

Input: Subject data (MRI, PET, tabular features)

Trained model $M^{*}$ with optimal hyperparameters $\Theta^{*}$

SHAP feature attribution function

Intervention mapping rules $R$

Output: Risk probabilities $p_{k}$ (4 classes), intervention recommendations I

1: Extract fused representation $z_{fused}\leftarrow M*\left( X_{MRI}, X_{PET}, X_{tab} \right)$

2: Compute risk probabilities $p_{k}$ for $k \in\left\{ Normal, SCD, MCR, Progression \right\}$

3: Compute intervention urgency score ← $sigmoid\left( FC\left( z_{fused} \right) \right)$

4: Estimate SHAP values $\varphi_{i}$ for each feature $i$

5: Identify top 5 features $\Phi_{top5}\leftarrow argsort\left( \left| \varphi_{i} \right|, descending \right)\left[ 1,\ldots,5 \right]$

6: Determine dominant risk class $k* \leftarrow argmax\left( p_{k} \right)$

7: If $\max\left( p_{k} \right)<0.5$: $\left\{ Conservative threshold \right\}$

8: Recommend "Continue monitoring" with annual follow-up

9: Else:

10: Lookup intervention rules $R\left( k*, \Phi_{top5} \right)$

11: Extract: recommended interventions, intensity level, monitoring frequency

12: I ← formatted clinical summary with confidence scores

13: End If

14: Return p, I (including SHAP-based feature explanation)
